# Sequence variation in the β7–β8 loop of bacterial class A sortase enzymes alters substrate selectivity

**DOI:** 10.1016/j.jbc.2021.100981

**Published:** 2021-07-22

**Authors:** Isabel M. Piper, Sarah A. Struyvenberg, Jordan D. Valgardson, D. Alex Johnson, Melody Gao, Katherine Johnston, Justin E. Svendsen, Hanna M. Kodama, Kelli L. Hvorecny, John M. Antos, Jeanine F. Amacher

**Affiliations:** 1 Department of Chemistry, Western Washington University, Bellingham, Washington, USA; 2 Department of Biochemistry, University of Washington, Seattle, Washington, USA

**Keywords:** sortases, enzymes, target selectivity, structural biology, protein biochemistry, protein engineering, Abz, 2-aminobenzoyl fluorophore, baSrtA, *Bacillus anthracis* SrtA, CWSS, cell wall sorting signal, Dnp, 2,4-dinitrophenyl quencher, lmSrtA, *Listeria monocytogenes* SrtA, saSrtA, sortase A from *Staphylococcus aureus*, SEC, size-exclusion chromatography, SML, sortase-mediated ligation, spSrtA, *Streptococcus pneumoniae* SrtA, spSrtA_faecalis_, β7–β8 loop residues from *Enterococcus faecalis*, spSrtA_lactis_, β7–β8 loop residues from *Lactococcus lactis*, spSrtA_monocytogenes_, β7–β8 loop residues from *Listeria monocytogenes*, spSrtA_oralis_, β7–β8 loop residues from *Streptococcus oralis*, spSrtA_suis_, β7–β8 loop residues from *Streptococcus suis*

## Abstract

Gram-positive bacteria contain sortase enzymes on their cell surfaces that catalyze transpeptidation reactions critical for proper cellular function. *In vitro*, sortases are used in sortase-mediated ligation (SML) reactions for a variety of protein engineering applications. Historically, sortase A from *Staphylococcus aureus* (saSrtA) has been the enzyme of choice to catalyze SML reactions. However, the stringent specificity of saSrtA for the LPXTG sequence motif limits its uses. Here, we describe the impact on substrate selectivity of a structurally conserved loop with a high degree of sequence variability in all classes of sortases. We investigate the contribution of this β7–β8 loop by designing and testing chimeric sortase enzymes. Our chimeras utilize natural sequence variation of class A sortases from eight species engineered into the SrtA sequence from *Streptococcus pneumoniae*. While some of these chimeric enzymes mimic the activity and selectivity of the WT protein from which the loop sequence was derived (*e.g.*, that of saSrtA), others results in chimeric *Streptococcus pneumoniae* SrtA enzymes that are able to accommodate a range of residues in the final position of the substrate motif (LPXTX). Using mutagenesis, structural comparisons, and sequence analyses, we identify three interactions facilitated by β7–β8 loop residues that appear to be broadly conserved or converged upon in class A sortase enzymes. These studies provide the foundation for a deeper understanding of sortase target selectivity and can expand the sortase toolbox for future SML applications.

Sortases are cysteine transpeptidase enzymes that gram-positive bacteria use to covalently attach proteins to their cell wall for various functions, including the assembly of pili or display of virulence factors (1, 2, 3). There are six recognized classes of sortase enzymes (classes A–F), with roles *in vivo* ranging from general purpose or “housekeeping” functions (classes A and E), to more specific roles such as the construction of the bacterial pilus (class C) (1, 4). These enzymes recognize a cell wall sorting signal (CWSS) on the outer membrane of gram-positive bacteria (1, 5). For class A sortases, the CWSS is the sequence LPXTG (1, 5). Using previously published numbering (L = P4, P = P3, X = P2, T = P1, and G = P1′), P4, P3, and/or P1′ of this motif vary among different classes (5). After target recognition, a His–Cys–Arg catalytic triad facilitates a transpeptidation reaction whereby the CWSS is first cleaved between the P1 and P1′ residues *via* nucleophilic attack by the catalytic Cys, resulting in a thioester linkage with the P1 position of the CWSS. Resolution of this acyl-enzyme intermediate is then achieved by nucleophilic attack by an amino group displayed on the cell wall building block lipid II, or in the case of pilus formation, displayed on a separate protein subunit (1, 3, 5, 6). The final result is the formation of a new amide linkage, with the portion of the substrate N-terminal to the CWSS now covalently attached at its C terminus to the amine nucleophile ligation partner.

The ability to cleave a substrate sequence and subsequently ligate a second component (for example a protein or synthetic peptide derivative) makes sortases an attractive tool for protein engineering efforts, commonly called sortase-mediated ligation (SML) or sortagging (3). Sortase A from *Staphylococcus aureus* (saSrtA) was the first of these enzymes discovered and continues to see widespread use for *in vitro* SML experiments (1, 7). Notable improvements in SML technology have occurred in recent years, including strategies for limiting the reversibility of the ligation reaction and the development of saSrtA variants with dramatically improved catalytic efficiency (3, 8, 9). However, as a consequence of the narrow substrate selectivity of saSrtA (10), the majority of SML examples rely on the combination of one ligation partner displaying an LPXTG motif near its C-terminus with another possessing one or more N-terminal glycines. This restricted substrate scope can be advantageous, for example, in the use of SML for labeling specific polypeptides in complex mixtures, but it also represents a limitation for certain applications (9, 11, 12). Highlighting this point, an increasing number of studies have demonstrated that the use of naturally occurring sortases or engineered sortases with altered substrate selectivity offers distinct advantages such as reducing the necessity for point mutations in protein semisynthesis applications (12), enabling the labeling of endogenous proteins that do not naturally contain the LPXTG motif (11, 13), and allowing labeling of multiple sites within the same protein target (11, 14). Thus, the engineering and discovery of sortases with altered substrate profiles, along with a better understanding of the biochemical basis for sortase substrate selectivity, represent important areas for the continued development of SML technology.

Previous mutagenesis and structural studies of various sortases provide a wealth of knowledge about substrate recognition, including initial ligand recognition and subsequent cleavage (thioesterification), as well as nucleophile recognition and mechanistic details of peptide ligation (transpeptidation) (1, 2, 15). Specifically, the catalytic residues of all native sortases identified to date are (using saSrtA numbering unless specified otherwise) as follows: His120 (general acid/base), Cys184 (nucleophile, acyl-enzyme intermediate), and Arg197 (transition state stabilization) (Fig. 1*A*) (1, 15). In addition, directed evolution studies have identified mutations (P94R/D160N/D165A/K190E/K196T) that are together able to boost the catalytic efficiency of saSrtA by 120-fold (8). Of these five mutations, several are in two of the three structurally conserved loops in class A sortases located near the peptide-binding cleft: those between the β4, β5 strands (β4–β5 loop), the β6, β7 strands (β6–β7 loop, where D165A occurs), and the β7, β8 strands (β7–β8 loop, where K190E and K196T are located). Notably, while the increase in enzyme activity afforded by these mutations included a 3.6-fold increase in *k*_cat_, the effect was dominated by a 33-fold decrease in *K*_M_, suggesting these loop residues may be important in CWSS recognition (8).

**Figure 1:**
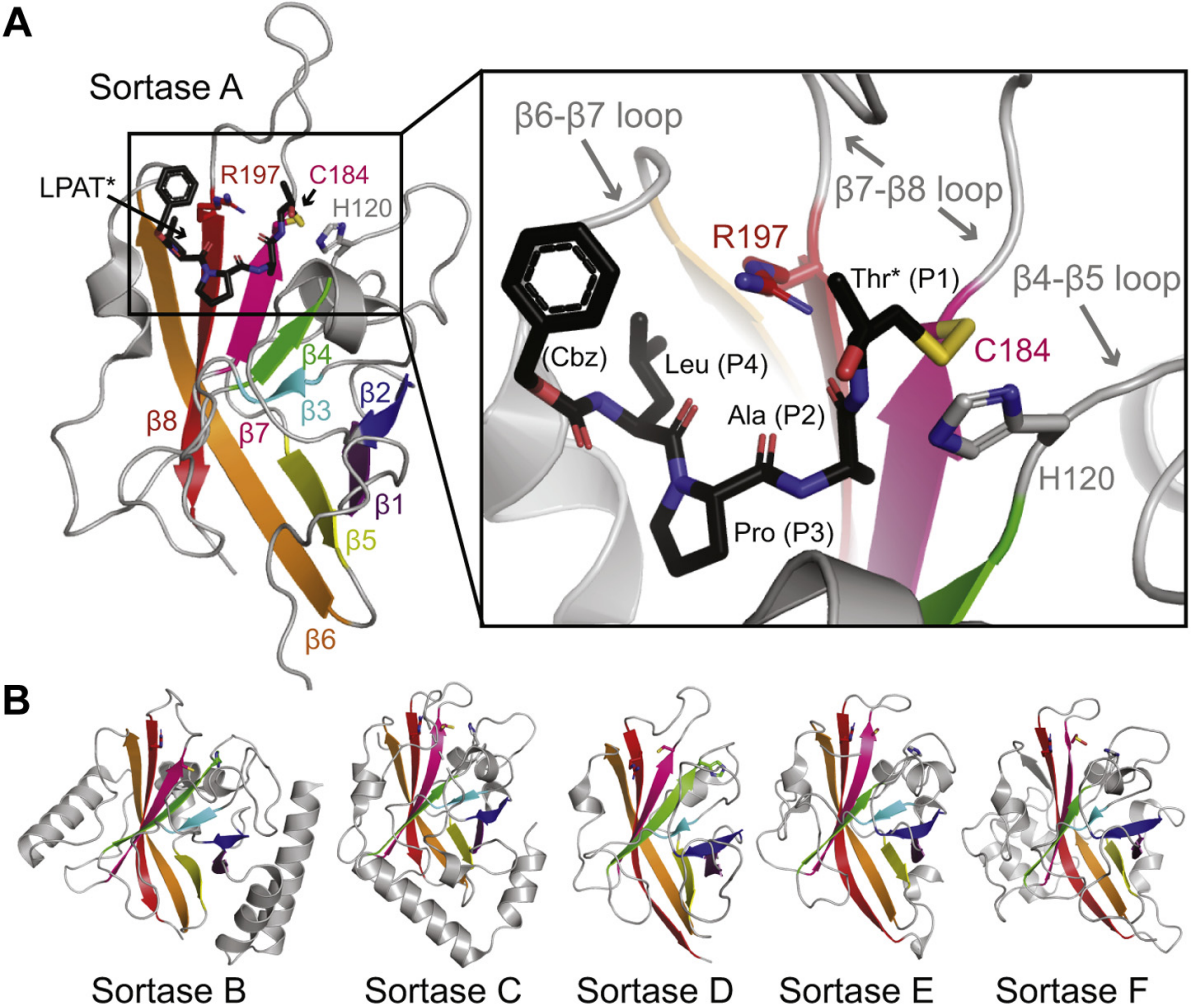
**The sortase-fold is conserved in all classes of bacterial sortases**. *A*, the peptide-bound structure of *S. aureus* SrtA (saSrtA) is shown in *cartoon* representation, with β-strands colored and labeled (PDB ID: 2KID) (19). The side chains of the catalytic residues (H120, C184, and R197) are shown as *sticks*, colored by heteroatom (O = *red*, N = *blue*, S = *yellow*), and labeled. The disulfide-linked peptide analog, Cbz-LPAT∗, where Cbz is a carbobenzyloxy protecting group and T∗ is (2*R*,3*S*)-3-amino-4-mercapto-2-butanol, is shown as *black sticks* and colored by heteroatom (19). A zoomed-in version of the active site is shown in the *black box*, with features indicated as in *panel A*. The variable loops are labeled and indicated by *gray arrows*. *B*, the overall sortase fold is well conserved in proteins of different classes. Here, structures for class B (PDB ID: 1NG5), class C (3O0P), class D (2LN7), class E (5CUW), and class F (5UUS) sortases are shown in *cartoons*, with conserved β-strands colored as in *panel A*, highlighting the 8-stranded sortase fold. The conserved catalytic triad is shown in *sticks* (and colored by heteroatom) for all.

Additional evidence for the role of loop residues was obtained from more targeted directed evolution and mutagenesis studies. For example, it was demonstrated that the β6–β7 loop of saSrtA directly confers specificity at P4 of the recognition motif (LPXTG), and residues other than leucine (L) can be accommodated using sortases with mutations in the β6–β7 loop (12, 16, 17). Indeed, substitution of the β6–β7 loop residues from saSrtB into the saSrtA enzyme alters substrate recognition to that of a sortase B protein (NPQTN) (18). Turning to the β7–β8 loop, the NMR structure of saSrtA covalently bound to a modified LPAT∗ peptide mimetic revealed a noncovalent interaction between W194 in saSrtA and the Thr residue in P1 (LPXTG) (19, 20). Mutation of W194 in saSrtA decreased the reaction rate, although it was not essential to catalysis (20). Taken together, these past studies reveal that sequence variation within sortase loops directly affects both activity and selectivity for target ligands. Furthermore, conservation of the closed eight-stranded β-barrel core in all sortase A-F structures that have been reported to date suggests that these principles may apply to non–class A sortases as well (Fig. 1*B*) (2).

In this work, we specifically look at natural sequence variation in the β7–β8 loop of class A sortases, using *Streptococcus pneumoniae* SrtA (spSrtA) as a model system. The β7–β8 loop was initially identified after sequence and evolutionary conservation analyses as a region of notable variability in class A (and other) sortases. We find that the β7–β8 loop sequence dramatically affects both overall enzyme activity and selectivity at P1′ of the CWSS. Our data are consistent with a recent publication that investigated the grafting of β7–β8 loop sequences from saSrtA and *Bacillus anthracis* SrtA (baSrtA) into *Streptococcus pyogenes* SrtA (18). This work also suggested that W194 (saSrtA numbering) may play a role in the substrate recognition of the reported chimeras (21). Here, we have profiled the substrate preferences of over a dozen loop chimeras and single- or double-mutants targeting the β7–β8 loop. While we also observe a role for W194 in substrate recognition, our data suggest that it is unique to saSrtA and not broadly applicable to describe β7–β8 loop–mediated class A sortase function. Indeed, the combination of functional enzyme assays and analysis of reported sortase structures in the present work suggests three different β7–β8 loop–mediated interactions that affect selectivity and activity.

## Results

### Sequence analyses of bacterial sortases

**Figure 2:**
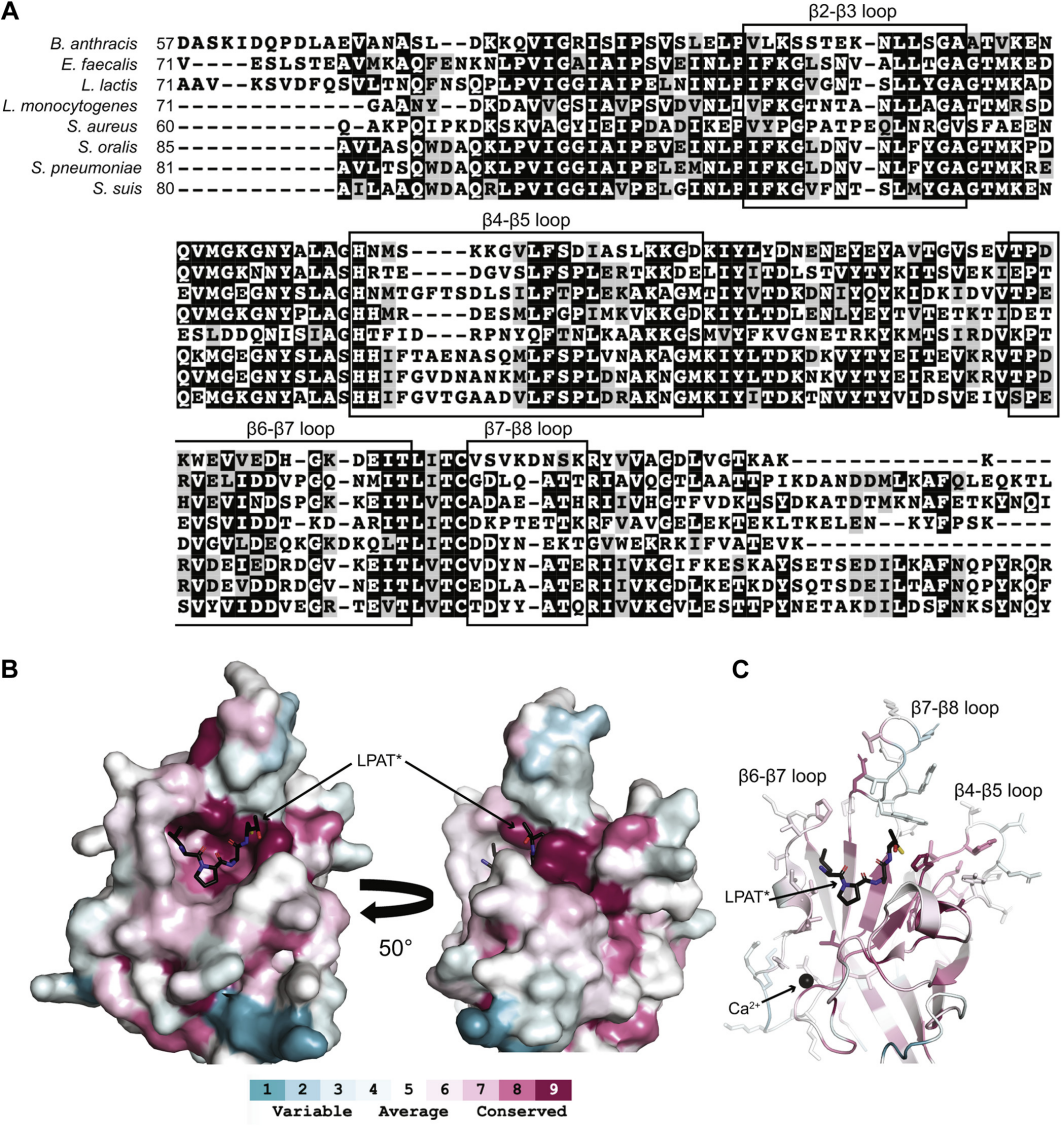
**Sequence analysis and evolutionary conservation of class A sortases**. *A*, multiple sequence alignment of previously studied class A sortase sequences (22). Alignment was performed using T-Coffee, and the figure was prepared using BoxShade. *Black boxes* indicate loop regions in saSrtA, as labeled. The *black box* for the β7–β8 loop cuts off the final five residues of the saSrtA loop (GVWEK) because the alignment is not correct in this region. *B*, the ConSurf server was used to investigate evolutionary conservation, using saSrtA (PDB ID: 2KID) as a template and a multiple sequence alignment of 400 class A sortase sequences. The results are shown on 2KID in surface representation (key below) and the LPAT∗ peptidomimetic is in *black sticks* and colored by heteroatom. Conservation is highest in the peptide-binding cleft and on the backside of the active site, where lipid II is hypothesized to bind. *C*, cartoon representation of the ConSurf results as described in panel B, with the side chain *sticks* of the β4–β5, β6–β7, and β7–β8 loops shown. SaSrtA binds calcium, which is shown as a *black sphere* and labeled. saSrtA, sortase A from *Staphylococcus aureus.*

To investigate general sequence variation in class A sortases, we first created a multiple sequence alignment of the eight SrtA sequences used in our previous work, proteins from: *B. anthracis*, *Enterococcus faecalis*, *Lactococcus lactis*, *Listeria monocytogenes*, *S. aureus*, *Streptococcus oralis*, *S. pneumoniae*, and *Streptococcus suis* (Fig. 2*A*) (22). Although this is a small subset of SrtA sequences, we reasoned that this representative group would reveal general sequence trends or sequence variations as these enzymes had exhibited clear differences in substrate preferences in our previous study (19). Indeed, variations were present in loop regions, specifically those of the β2–β3, β4–β5, β6–β7, and β7–β8 loops (Fig. 2*A*).

In addition to our sequence analysis, we also wanted to look more broadly at global evolutionary conservation in class A sortases. We extracted 400 SrtA sequences from the NCBI and used MAFFT to create a multiple sequence alignment (23). To visualize evolutionary conservation, we used the ConSurf server, with saSrtA (PDB ID: 2KID) as our structural template (Fig. 2*B*) (24, 25). This analysis confirmed that class A sortase sequences are quite variable, with very few residues showing a high degree of conservation (dark maroon color). However, validating our analysis, the peptide-binding cleft (occupied by the peptidomimetic LPAT∗ in 2KID) is very well conserved, as is the presumed endogenous lipid II–binding site on the backside of the enzyme (Fig. 2*B*) (2, 26).

As suggested by our initial multiple sequence alignment (Fig. 2*A*), ConSurf confirmed that the three structural loops that border the peptide binding cleft, the β4–β5, β6–β7, and β7–β8 loops, are all quite variable (Fig. 2*C*). Any conservation in these loops that we do see may also be an artifact of the multiple sequence alignment because of variable lengths; for example, E195 saSrtA in the β7–β8 loop appears to be highly conserved, but this is because of the fact that out of the 400 sequences used in the alignment, only 10 have any residue at this position (9 of which are Glu) and all are of *Staphylococcus* sequences.

Given that the β6–β7 loop has been shown to be intimately involved in sortase substrate recognition, we were intrigued that our analysis revealed similar levels of variability in the β4–β5 and β7–β8 loops (18). In the case of β7–β8, we were also motivated by its proximity to the enzyme active site and CWSS P1′ position (15). Therefore, we sought to further explore how the β7–β8 loop affects the activity and substrate specificity of a sortase with narrow substrate tolerance (saSrtA) *versus* one that is more promiscuous (spSrtA).

### Loop-swapped β7–β8 variants reveal differences in position P1′ selectivity for *S. aureus* and *S. pneumoniae* SrtA enzymes

In our previous work, we found that the most striking differences in substrate tolerances among the class A sortases studied were observed at the P1′ (LPXTG) of the substrate motif (19). For example, while saSrtA is specific for a Gly residue at P1′, SrtA from *S. pneumoniae* (spSrtA) recognizes over 10 of the 20 amino acids at this position in a 24-h end-point assay (22). To determine whether the β7–β8 loop played a role in these differing substrate preferences, we began by engineering two loop-swapped variants: saSrtA_pneumoniae_ (which contains the β7–β8 loop residues from spSrtA (CEDLAATER, where the catalytic cysteine and arginine are underlined)) and spSrtA_aureus_ (with β7–β8 residues CDDYNEKTGVWEKR from saSrtA). Notably, the length of the saSrtA β7–β8 loop contains an additional five residues, as compared with the spSrtA β7–β8 loop. The saSrtA loop also uniquely contains W194, which is predicted to directly contact the P1 threonine of the LPXTG motif (19). In addition, while both loops are predicted to have an overall net negative charge at the physiological pH, the saSrtA loop contains two positively charged lysine residues that are not present in spSrtA. Both chimeric sortases were expressed and purified from *Escherichia coli* and were isolated as soluble, monomeric enzymes as described previously and in Experimental procedures (Fig. S1) (22). Based on the migration of these variants using size-exclusion chromatography (SEC), the variants are not aggregated and retain a similar radius of gyration and oligomeric status (Fig. S1).

**Figure 3:**
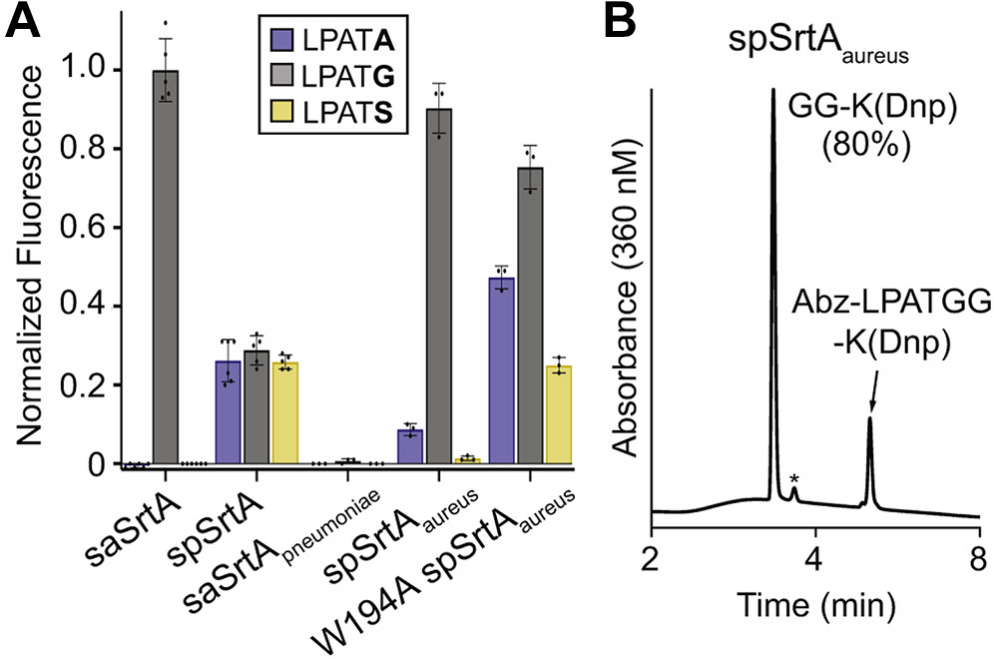
**Interchanging β7–β8 loops in class A sortases modulates substrate selectivity and activity for target sequences that vary at position P1′ of the canonical LPXTG motif**. *A*, comparison of substrate selectivity for WT saSrtA and spSrtA proteins, as well as β7–β8 loop chimeras saSrtA_pneumoniae_, spSrtA_aureus_, and W194A spSrtA_aureus_. Substrate cleavage was monitored *via* an increase in fluorescence at 420 nm from reactions of the fluorophore-quencher probes Abz-LPATGG-K(Dnp), Abz-LPATAG-K(Dnp), and Abz-LPATSG-K(Dnp) (represented as LPAT**G**, LPAT**A**, and LPAT**S**) in the presence of excess hydroxylamine. Bar graphs represent the mean normalized fluorescence (± SD) from at least three independent experiments at the 2-h reaction timepoint, as compared with saSrtA and the peptide LPAT**G**. *B*, representative HPLC chromatogram for the reaction of Abz-LPATGG-K(Dnp) and H_2_NOH in the presence of spSrtA_aureus_. This reaction was conducted in the presence of Ca^2+^. Selective cleavage between the threonine (T) and glycine (**G**) residues was observed, with an overall conversion of 80% (∗ = Abz-LPAT-NHOH reaction product. Low peak intensity is due to the weak absorbance of Abz at 360 nm). Additional HPLC data for select substrate/enzyme pairings are provided in Figure S2, *B–F*. saSrtA, sortase A from *Staphylococcus aureus*; spSrtA_monocytogenes_, β7–β8 loop residues from *Listeria monocytogenes*; Abz, 2-aminobenzoyl fluorophore; Dnp, 2,4-dinitrophenyl quencher.

To monitor enzymatic activity and selectivity of the saSrtA_pneumoniae_, spSrtA_aureus_, and their WT counterpart proteins, we utilized well-established FRET quencher probes consisting of different substrate motifs flanked by a 2-aminobenzoyl fluorophore (Abz) and a 2,4-dinitrophenyl quencher (Dnp) (20, 27, 28). Probes containing three substrate variants were initially prepared (Abz-LPAT**A**G-K(Dnp), Abz-LPAT**G**G-K(Dnp), Abz-LPAT**S**G-K(Dnp), varying only at P1′ in bold) and used to test the relative activity of our WT and chimeric enzymes. For simplicity, we have hereafter omitted the Abz, K(Dnp), and C-terminal glycine from peptide descriptions. For comparing enzyme activity, a standard 2-h reaction time was utilized and an excess of H_2_NOH was included to resolve the acyl enzyme intermediates. For consistency, all reactions were also conducted in the presence of Ca^2+^, which is a required cofactor for saSrtA. A reaction end point (indicated by the increase in Abz fluorescence) for all enzyme/substrate pairings was then expressed relative to averaged benchmark reactions of WT saSrtA with the standard LPAT**G** substrate (Figs. 3*A* and S2*A*). This benchmark reaction was consistently found to give ∼84% conversion to the expected transacylation products when independently monitored *via* RP-HPLC (Fig. S2*B*).

Based on our previous results, we predicted that spSrtA would show activity for all three peptides, while saSrtA would be selective for LPAT**G** (22). Consistent with this prediction, our results confirmed that spSrtA was equally capable of processing all three substrates, whereas saSrtA was restricted to LPAT**G** (Fig. 3*A*). Our assay also revealed a marked reduction in spSrtA activity *versus* saSrtA, which was not captured in our previous study, likely because of the extended reaction time (24 h) used in that work (22). With respect to the chimeric enzymes, our results clearly showed that the sequence of the β7–β8 loop was a major determinant of activity and specificity. Specifically, the saSrtA_pneumoniae_ protein was completely inactive while spSrtA_aureus_ functionally mimicked the narrow substrate preference of the WT saSrtA enzyme (Fig. 3*A*). This result is consistent with recently published data (21). To verify that our sortases were cleaving substrates at the expected site, reactions exhibiting a normalized fluorescence value of 0.2 or higher were independently monitored by RP-HPLC and LC-MS, which confirmed cleavage between P1 and P1′ (Figs. 3*B* and S2, *B–F*, Table S1). Notably, reactions for HPLC and LC-MS characterization were conducted in the presence and absence of Ca^2+^, which demonstrated that this cofactor was not required for the activity of spSrtA and spSrtA_aureus_.

Continuing on with the SpSrtA_aureus_ chimera, we next wanted to determine if the Trp residue derived from the saSrtA loop played a significant role in enzyme activity. In WT saSrtA, the W194 residue (using saSrtA numbering) is known to affect enzyme activity of saSrtA and has been shown to interact with the threonine of the LPXTG motif (Fig. S3) (19, 20). We therefore expressed and purified the corresponding “W194A” mutant of spSrtA_aureus_ and tested this variant with A-, G-, and S-containing peptides in our assay. This mutation in saSrtA has previously been characterized with respect to enzymatic activity but was not previously investigated with respect to possible effects on P1′ selectivity (20). Indeed, our W194A spSrtA_aureus_ protein exhibited a 17% reduction in reaction progress for LPAT**G**, while retaining its preference for Gly-containing peptides (Fig. 3*A*). However, W194A spSrtA_aureus_ also revealed activity for the A- and S-containing peptides suggesting that the Trp residue acts as a selectivity filter (Fig. 3*A*).

### Variability in position P1′ selectivity and transpeptidase activity in *S. pneumoniae* SrtA β7–β8 variants

**Figure 4:**
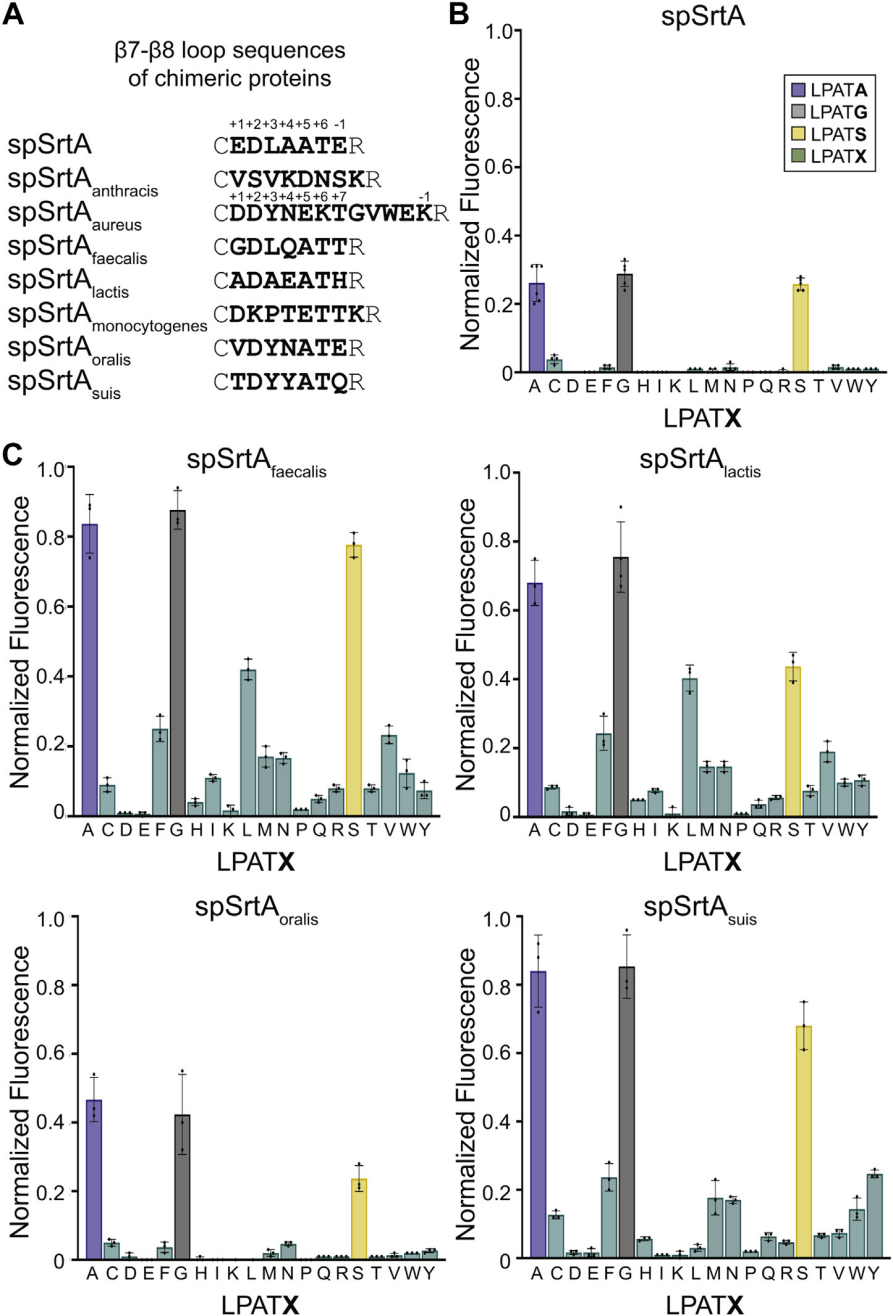
**The sequence of the β7–β8 loop dramatically affects selectivity and activity for spSrtA**. *A*, the β7–β8 loop sequences of the chimeric proteins used are listed, with representative numbering for residues in the β7–β8 loop labeled for spSrtA and spSrtA_aureus_. *B* and *C*, substrate selectivity profiles for WT spSrtA (B) and chimeric spSrtA variants (*C*). Substrate cleavage monitored *via* an increase in fluorescence at 420 nm from reactions of fluorophore-quencher probes with the generic structure Abz-LPATXG-K(Dnp) (LPAT**X**) in the presence of hydroxylamine. Bar graphs represent the mean normalized fluorescence (±SD) from at least three independent experiments. spSrtA_faecalis_, spSrtA with the β7-β8 loop residues from *Enterococcus faecalis*; spSrtA_lactis_, spSrtA with the β7-β8 loop residues from *Lactococcus lactis*; spSrtA_oralis_, spSrtA with the β7-β8 loop residues from *Streptococcus oralis*; spSrtA_suis_, spSrtA with the β7-β8 loop residues from *Streptococcus suis*; Abz, 2-aminobenzoyl fluorophore; Dnp, 2,4-dinitrophenyl quencher.

In addition to the profound shift in substrate scope observed for spSrtA_aureus_, we were also intrigued that the overall reactivity of this chimera for LPAT**G** was comparable with that seen with WT saSrtA. This stood in sharp contrast to the reaction of LPAT**G** with WT spSrtA, where reaction progress was nearly two-thirds lower within the 2-h reaction time of our assay (Fig. 3*A*). Based on this, we wondered if similar gains in reactivity for substrates other than LPAT**G** could be achieved by substituting in residues from additional SrtA proteins (22, 27). To test this, we created an additional six spSrtA variants containing loop residues from SrtA proteins that we had evaluated previously (22). These chimeras included the β7–β8 loop residues from *B. anthracis*, *E. faecalis* (spSrtA_faecalis_), *L. lactis* (spSrtA_lactis_), *L. monocytogenes* (spSrtA_monocytogenes_), *S. oralis* (spSrtA_oralis_), and *S. suis* (spSrtA_suis_) (Fig. 4*A*) (22). To avoid confusion in the numbering of loops with variable lengths, we will hereafter refer to the N-terminal positions of each β7–β8 loop by numbering with respect to the catalytic Cys (β7–β8^+1^, β7–β8^+2^, etc.) that precedes the loop, whereas the C-terminal loop residue will be numbered relative to the catalytic Arg (β7–β8^−1^) (Fig. 4*A*).

The six chimeric proteins were expressed and purified using the same protocol as spSrtA_aureus_, and as described in Experimental procedures. The purity of all proteins was validated by SDS-PAGE, and SEC was consistent with the isolated proteins being predominantly monomeric (Fig. S1). With the new chimeras in hand, we conducted an initial evaluation of relative activity using the LPAT**G**, LPAT**A**, and LPAT**S** substrates described above. While the majority of constructs exhibited significant reactivity across all three substrates, spSrtA_anthracis_ and spSrtA_monocytogenes_, containing the β7–β8 loop residues from *B. anthracis* and *L. monocytogenes*, respectively, proved to be mostly inactive (Fig. S4*A*). For the remaining enzymes, the spSrtA_oralis_ protein behaved similarly to WT spSrtA, while spSrtA_faecalis_, spSrtA_lactis_, and spSrtA_suis_ showed improved performance for A-, G-, and S-containing substrates (Fig. 4, *B* and *C* and S4*A*). This was particularly interesting in the case of spSrtA_faecalis_, given that the WT SrtA enzyme from *E. faecalis* was previously shown to have poor reactivity for the same test substrates despite the use of higher enzyme loading and considerably longer reaction times (22).

Based on initial experiments with the A-, G-, and S-containing peptides, we next wanted to expand our peptide pool to assess the relative reactivity of our active chimeric spSrtA variants for peptides containing all 20 amino acids at P1′. For comparison, a similar substrate profile was generated for WT spSrtA. As shown in Figure 4*B*, within the 2-h time frame of our assay, the WT protein was rather selective in its substrate recognition, with reactivity limited to A-, G-, and S-containing peptides. We note here that this somewhat limited substrate scope that appears to differ from the more promiscuous behavior reported previously for spSrtA. We attribute this to the fact that longer reaction times (24 h) and higher enzyme loadings (5-fold higher than the loading used here) were utilized in this earlier work (22). Similar to WT spSrtA, the spSrtA_oralis_ was limited to A-, G-, and S-containing peptides, albeit with slightly elevated reactivity in the case of LPAT**A** and LPAT**G**. Finally, we were intrigued to find that our spSrtA_faecalis_, spSrtA_lactis_, and spSrtA_suis_ proteins all show increased promiscuity for a variety of amino acids at P1′ in our assay (Fig. 4*C*).

Overall, the spSrtA_faecalis_, spSrtA_lactis_, and spSrtA_suis_ proteins showed the largest increase in activity and promiscuity for this library of peptides. The spSrtA_faecalis_ and spSrtA_lactis_ proteins each recognized 15 of the 20 amino acids at P1′ with normalized fluorescence values of ≥0.05, while spSrtA_suis_ recognized 14 of the 20 (Fig. 4*C*). We chose 0.05 as a cut-off value to compare with the peptide activities of the spSrtA protein, which shows normalized fluorescence values of -0.02 to 0.02 for all non-G-, S-, or A-containing peptides, with the exception of LPAT**C** (at 0.04 ± 0.01). Furthermore, spSrtA_faecalis_ and spSrtA_suis_ exhibited ∼3-fold higher reaction progress for the G-, S-, and A-containing peptides than spSrtA.

As verification of the results of our fluorescence assay, we also characterized a subset of enzyme/substrate combinations using RP-HPLC and LC-MS. Focusing on spSrtA_faecalis_, we repeated reactions that exhibited normalized fluorescence values of ≥0.1 (LPAT**X**, **X** = A, F, G, I, L, M, N, S, V, W, Y) (Fig. S4*B*). Reactions were conducted in the absence of Ca^2+^ to confirm that this cofactor was not required for activity. Successful substrate cleavage was observed in all cases, ranging from a high of 78% conversion in the case of LPAT**G**, to only 6% conversion in the case of LPAT**W** over 2 h at room temperature (RT) (Fig. S4*B*). Notably, the trends in relative substrate preferences observed by HPLC were consistent with those found in our original fluorescence assay (Fig. S5). In addition, while LC-MS characterization confirmed that substrate cleavage was occurring between the P1 and P1′ of all sequences, certain substrates (LPAT**X**, **X** = W,F,L,M,Y) containing bulky hydrophobic residues also produced alternate products arising from cleavage on the C-terminal side of P1′ (Table S1, Fig. S4). In the case of LPAT**L**, this alternate cleavage product was actually the major species obtained after reaction with spSrtA_faecalis_. We note here that this capacity for alternate cleavage has been reported previously for WT spSrtA and thus appears to be maintained in the spSrtA_faecalis_ chimera (22).

### Variability in position P1′ selectivity and ligase activity in *S. pneumoniae* SrtA β7–β8 variants

**Figure 5:**
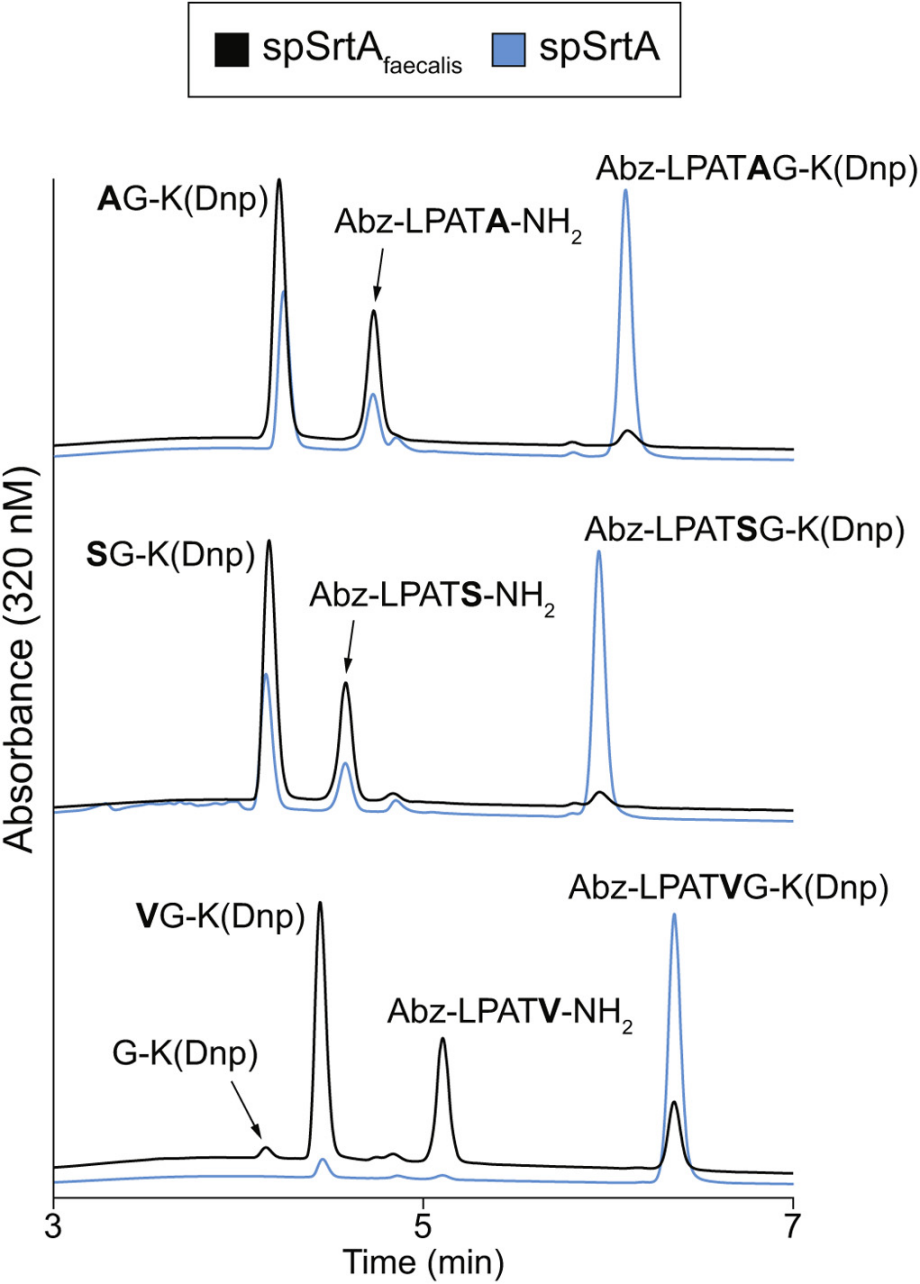
**spSrtA**_**faecalis**_**outperforms** WT **spSrtA in model amino acid ligation reactions**. HPLC chromatograms (320 nm) for model ligations between Abz-LPATXG-K(Dnp) and excess X-NH_2_ nucleophiles catalyzed by spSrtA_faecalis_ (*black curves*) or WT spSrtA (*blue curves*). Ligations were conducted in the absence of Ca^2+^. Chromatograms for LPAT**A**/**S** represent the 3-h reaction timepoint, and chromatograms for LPAT**V** correspond to the 8-h timepoint. All peak identities were confirmed *via* LC-MS (Table S1). spSrtA_faecalis_, spSrtA with the β7–β8 loop residues from *Enterococcus faecalis*; Abz, 2-aminobenzoyl fluorophore; Dnp, 2,4-dinitrophenyl quencher.

As a final assessment of the reactivity of the spSrtA_faecalis_ chimera, we next evaluated its ability to ligate amino acid nucleophiles in place of the H_2_NOH that was utilized in our fluorescence assay. For a series of test substrates (LPAT**X**, **X** = A,S,V), spSrtA_faecalis_ was able to successfully ligate the corresponding free amino acid carboxamides (**X**-NH_2_ = A-NH_2_, S-NH_2_, V-NH_2_) with very good efficiency (Fig. 5). As expected from our fluorescence assay results, reaction progress with LPAT**V** was slower than that observed for LPAT**A** and LPAT**S**. Specifically, reactions with LPAT**V** required 8 h at RT to consume 85% of the initial peptide substrate, whereas reactions with LPAT**A**/**S** exhibited >95% substrate conversion within 3 h. Importantly, the desired LPAT**X**-NH_2_ species was the major ligation product in all reactions as determined by LC-MS (Fig. 5, Table S1). Trace levels of substrate hydrolysis were also observed *via* LC-MS; however, the ratio of successful ligation to hydrolysis was 15:1 or better as estimated from mass spectral peak intensities. In reactions involving LPAT**V**, we also detected low levels of substrate cleavage on the C-terminal side of the P1′ valine residue. The extent of this alternate cleavage pathway was minimal, accounting for only ∼4% of the substrate cleavage events based on comparisons of HPLC peak areas for G-K(Dnp) and **V**G-K(Dnp) (Fig. 5). Interestingly, LC-MS characterization of these same reactions involving LPAT**V**, **V**-NH_2_, and spSrtA_faecalis_ failed to show clear evidence for the formation of ligation or hydrolysis products derived from the alternate cleavage pathway, potentially because of their low levels in solution.

For comparison, we also performed the same set of test ligations with WT spSrtA. In all cases, reaction progress was significantly reduced as compared with that of spSrtA_faecalis_ (Fig. 5). In particular, spSrtA exhibited minimal product formation with the LPAT**V** system, representing a 10-fold reduction in reaction progress relative to spSrtA_faecalis_ for this atypical sortase substrate motif. Building from this result, an initial attempt to utilize the LPAT**V** sequence as a handle for site-specific protein modification was made by installing this motif at the C-terminus of a full-size protein target. However, this protein substrate proved to be unreactive in the presence of both spSrtA_faecalis_ and WT spSrtA (data not shown).

### Mutagenic investigation of the contribution of β7–β8 loop residues

**Figure 6:**
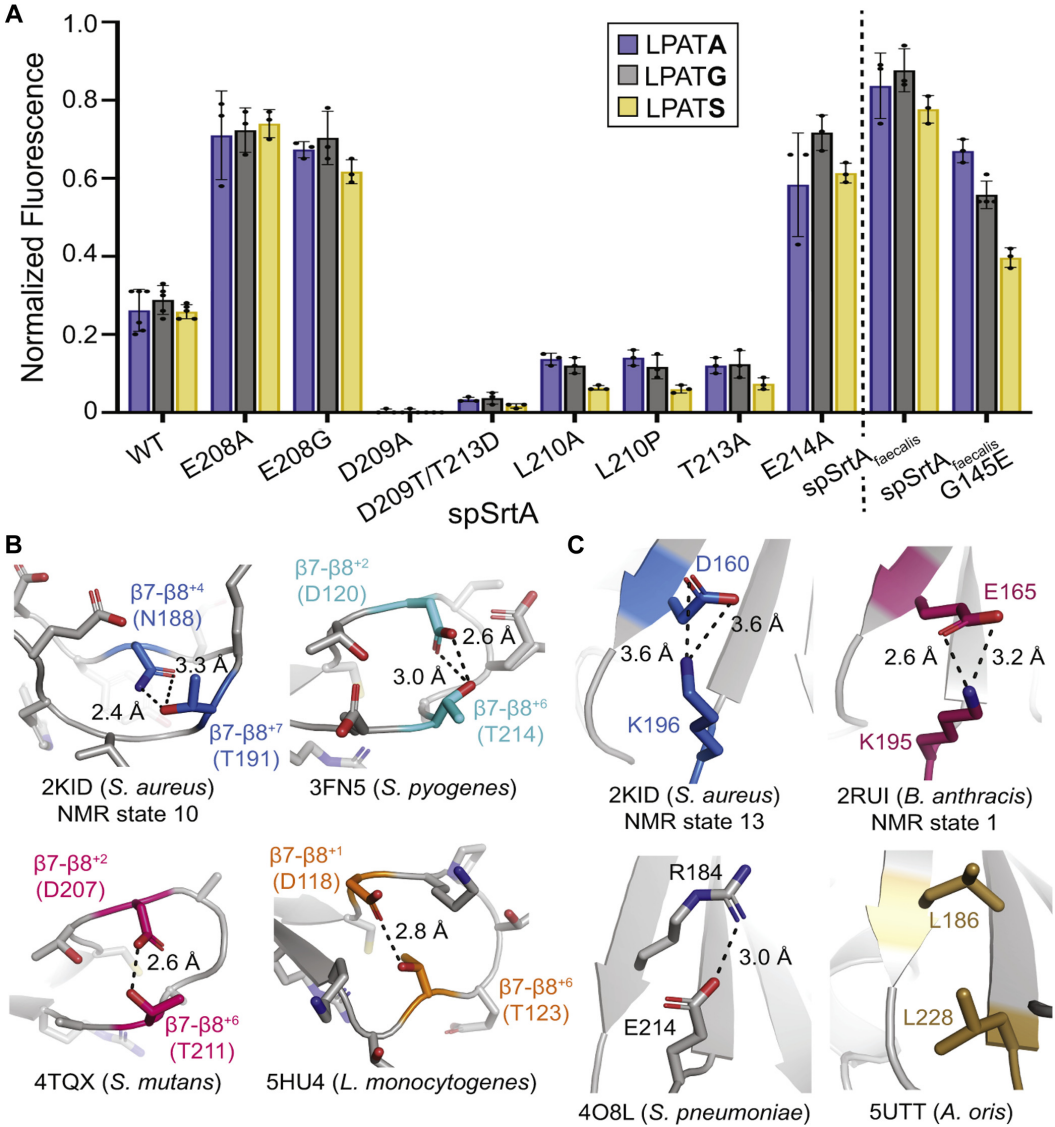
**Residues in the β7–β8 loop participate in interactions that affect enzyme activity and selectivity, based on structural analyses and mutagenesis**. *A*, enzyme assays on mutant spSrtA and spSrtA_faecalis_ variants (fluorophore-quencher substrate assay conditions identical to those described in the legend to Fig. 3). Bar graphs represent the mean normalized fluorescence values (± SD) from at least three independent experiments. *B* and *C*, all SrtA structures are in *gray ribbon*s. The β7–β8 loop side chains are all in *stick* representation and colored by heteroatom. Residues that participate in hydrogen bonds have non–*gray* carbons and are labeled. The hydrogen bonds are shown with a *black dashed line*, with measurements indicated. For NMR structures (PDB IDs 2KID and 2RUI), the state that was used for the image is labeled. Although not all NMR states contained the interaction indicated, in both cases, there were several states that revealed measurements consistent with a noncovalent interaction. Any side-chain *sticks* are colored by heteroatom (O = *red*, N = *blue*). *B*, β7–β8 intraloop hydrogen bond. The structures in this figure are as follows: *S. aureus* SrtA (2KID, *blue* carbons), *S. pyogenes* SrtA (3FN5, *cyan* carbons), *S. mutans* SrtA (4TQX, *pink* carbons), and *L. monocytogenes* SrtA (5HU4, *orange* carbons). *C*, interaction between the β7–β8 loop and β6 strand. The structures in this figure are as follows: *S. aureus* SrtA (PDB ID 2KID, *blue* carbons), *B. anthracis* SrtA (2RUI, *dark pink* carbons), *S. pneumoniae* SrtA (4O8L, *gray* carbons), and *A. oris* SrtA (5UTT, *gold* carbons). spSrtA_faecalis_, spSrtA with the β7–β8 loop residues from *Enterococcus faecalis*.

To dissect the contribution of each residue in the β7–β8 loop of spSrtA, we made a series of alanine mutations. Specifically, we mutated all non-Ala residues to alanine to produce the following spSrtA variants: E208A, D209A, L210A, T213A, and E214A. Analytical SEC of the final protein preparations suggested that these mutants were predominantly monomeric (Fig. S6). When tested using our FRET-based activity assay, we did not see major effects on P1′ selectivity in these mutants for G-, S-, and A-containing peptides; however, we did observe striking effects on overall reactivity (Fig. 6*A*). Specifically, a 2-fold increase in reaction progress was observed for the E208A and E214A mutations, suggesting that the native glutamic acid residues in spSrtA have a negative effect on its activity. The L210A and T213A mutations resulted in ∼50% reduction in reaction conversion, and the D209A spSrtA protein was entirely unreactive (Fig. 6*A*).

### Stereochemical basis of β7–β8 variant selectivity and activity—(1) A stabilizing intraloop hydrogen bond

To gain a stereochemical understanding of our biochemical results, we analyzed available structures of class A sortases in the Protein Data Bank. To our knowledge, the 3D structure of an active, monomeric form of spSrtA has yet to be reported. Available crystal structures of the domain-swapped dimer show that the β7–β8 loop is located at, and participates in, the dimer interface (PDB codes 4O8L, 4O8T, and 5DV0). Therefore, we chose to broaden our search to non-spSrtA structures, and in doing so, we identified three putative β7–β8 loop-mediated interactions in class A sortases, which will be described in the following three sections.

First, we observed an intra-loop hydrogen bond in the β7–β8 loops of several class A sortases (Fig. 6*B*). This Thr-mediated hydrogen bond is evident in SrtA proteins from *S. aureus* (PDB code 2KID), *S. pyogenes* (3FN5), *Streptococcus mutans* (4TQX), and *L. monocytogenes* SrtA (lmSrtA) (5HU4), among others not shown (Fig. 6*B*). Notably, the *S. pyogenes* SrtA protein (3FN5) includes multiple rotamers in one protomer at the Thr position (T214), and the hydrogen bond with D210 is conserved in both (Fig. S7*A*). We predict that in spSrtA, this hydrogen bond will be formed between the side chains of residues β7–β8^+2^ D209 and β7–β8^+6^ T213. Indeed, we see this interaction in a homology model of the monomeric spSrtA protein generated using SWISS-MODEL (Fig. S7*B*).

For this model, the *S. pyogenes* SrtA structure (PDB code 3FN5) was used as a template because the crystallized form of this enzyme (*S. pyogenes* SrtA residues S81-T249) has 63% sequence identity with spSrtA (29, 30, 31). An alignment of our spSrtA model with 3FN5 revealed an overall RMSD of 0.083 Å over 567 main chain atoms. We further validated our model using structural alignments with a monomer extracted from the domain-swapped dimer structure (RMSD of 0.603 Å over 483 main chain atoms), as well as other SrtA structures from *Streptococcus* species, including those from *Streptococcus agalactiae* and *S. mutans* (PDB codes 3RCC (RMSD of 0.773 Å over 384 main chain atoms) and 4TQX (RMSD of 0.456 Å over 530 main chain atoms)), respectively (Fig. S7, *C* and *D*).

Our mutagenesis results are consistent with this proposed interaction. Specifically, the T213A mutation, which disrupts a potential intraloop hydrogen bond between the β7–β8^+2^ D209 and β7–β8^+6^ T213, reduced spSrtA activity by 54 to 73% for G-, S-, and A-containing peptides (Fig. 6*A*). When we attempted to reverse the hydrogen bond geometry with a D209T/T213D double mutant, the resulting enzyme exhibited only trace reactivity (Fig. 6*A*). Taken together, our findings are consistent with at least one stabilizing intraloop hydrogen bond being a generally conserved and functionally relevant feature of β7–β8 SrtA loops. It should be noted, however, that the exact nature and location of this interaction likely varies. For example, in *Actinomyces oris* SrtA, this hydrogen bond is observed between β7–β8^+3^ D227 and β7–β8^+11^ S219 (Fig. S7*E*).

### Stereochemical basis of β7–β8 variant selectivity and activity—(2) A noncovalent interaction between the β6^−2^ residue and β7–β8 loop

In addition to an intraloop hydrogen bond, we observe interactions between the β7–β8 loop and β6^−2^ residues in multiple class A sortase structures (Fig. 6*C*). For example, K196 (β7–β8^−1^) of saSrtA interacts with the β6^−2^ D160 in several of the states of the reported NMR structure, PDB code: 2KID (Fig. 6*C*) (19). We also see a reasonable electrostatic interaction distance for the β7–β8^−1^ and β6^−2^ residues (K195 and E165, respectively) in several of the NMR states for baSrtA (PDB code 2RUI), as well as in the domain-swapped dimer structure of spSrtA (PDB code: 4O8L), which shows the E214 β7–β8 loop residue of one protomer interacting with R184 (Fig. 6*C*). Interestingly, the nature of this interaction can change, as in the case of *Actinomyces oris* SrtA, where both residues are hydrophobic leucine residues (L186 and L228 for the β6^−2^ and β7–β8^−1^ residues, respectively) (Fig. 6*C*).

Our results suggest that this β7–β8 loop/β6^−2^ interaction has a negative effect on sortase activity. This is supported by the >2-fold increase in substrate conversion for both the β7–β8^−1^ E214A and β7–β8^+1^ E208A spSrtA mutants as compared with WT spSrtA (Fig. 6*A*). While the reported domain-swapped dimer structure of spSrtA does not exhibit an obvious interaction between β7–β8^+1^ (E208) and β6^−2^ (R184), we note our spSrtA homology model does suggest this interaction is likely, with a distance between a guanidinium nitrogen atom of R184 and a side-chain carboxylate oxygen atom on E208 equal to 2.7 Å (Fig. S7*B*). As further evidence, given that this β7–β8^+1^ position is a glycine in the more active spSrtA_faecalis_ chimera, we expressed, purified, and tested the activity of two contrasting mutants at this site: E208G spSrtA (which would eliminate the putative β7–β8^+1^/β6^−2^ interaction) and G145E spSrtA_faecalis_ (*E. faecalis* SrtA numbering, which would restore the putative β7–β8^+1^–β6^−2^ interaction). Our results are consistent with our predictions, and we saw a 21 to 49% reduction in substrate conversion for the reaction of G-, S-, and A-containing peptides with G145E spSrtA_faecalis_ as compared with the initial spSrtA_faecalis_ chimera (Fig. 6*A*). In contrast, a >2-fold increase in reaction progress was observed for E208G spSrtA relative to WT spSrtA (Fig. 6*A*).

Overall, our data are consistent with recent work where a triple mutant of *S. pyogenes* SrtA (E189H/V206I/E215A, where E215A is a mutation at the β7–β8^−1^ position) resulted in 6.6-fold enhanced catalytic efficiency (32). In addition, K196T in the catalytically enhanced pentamutant saSrtA protein is also located at the β7–β8^−1^ position (8). Taken together, the data support that a noncovalent interaction between the β7–β8 loop and β6 strand negatively affects SrtA activity.

### Stereochemical basis of *β*7–*β*8 variant selectivity and activity—(3) An interaction between the *β*7–*β*8 and *β*4–*β*5 loops

**Figure 7:**
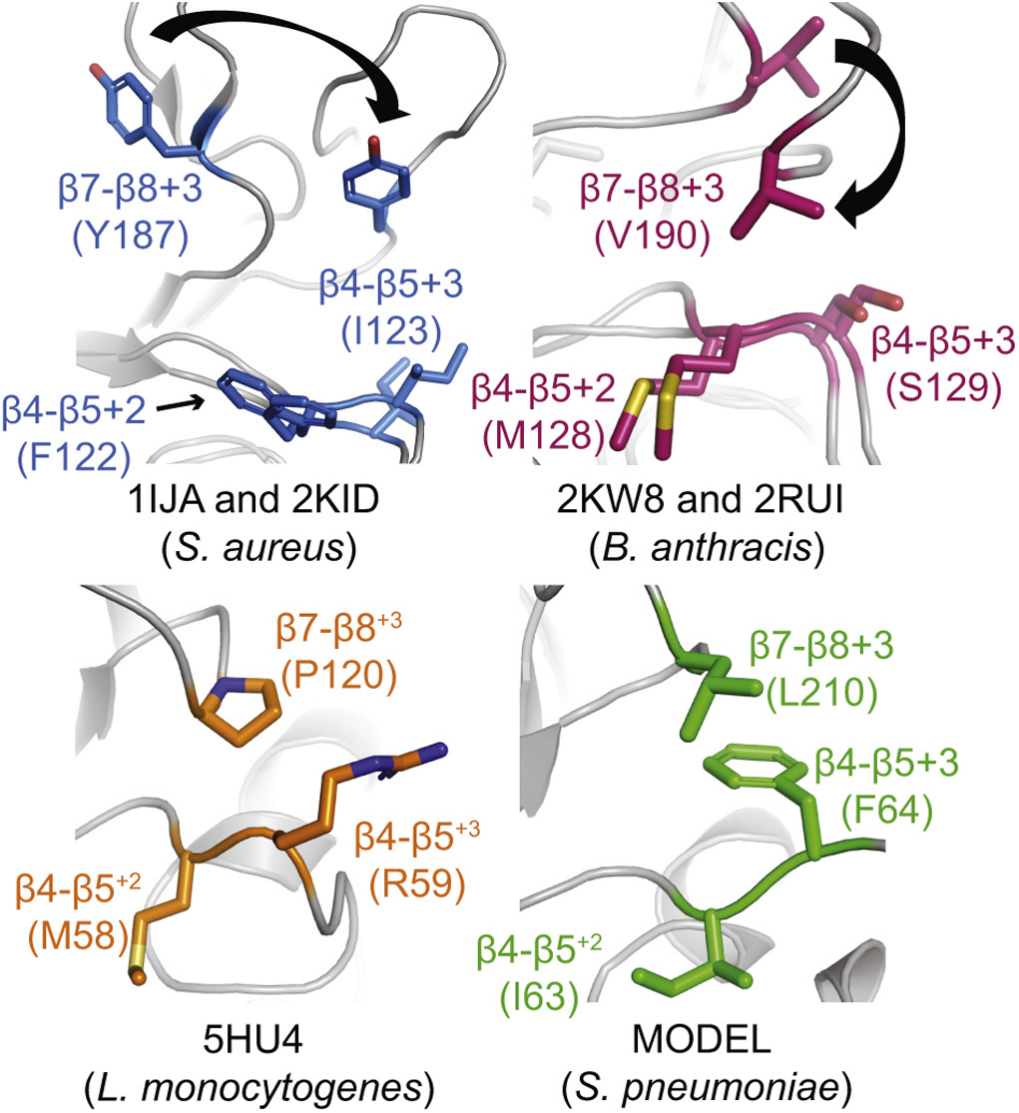
**Interaction between the β7–β8 and β4–β5 loops**. All SrtA structures are in *gray ribbon*s. The β7–β8 loop side chains are all in *stick* representation and colored by heteroatom. The structures in this figure are as follows: *S. aureus* (unbound: 1IJA and bound: 2KID, *blue* carbons), *B. anthracis* (unbound: 2KW8 and bound: 2RUI, *dark pink* carbons), *L. monocytogenes* (5HU4, *orange* carbons), and *Streptococcus pneumoniae* (homology model (Fig. S7), *green* carbons). The *arrows* indicate residue movement from the unbound to bound structures.

NMR structures of saSrtA and baSrtA proteins in the unbound and bound states (PDB codes: 1IJA, 2KID, 2KW8, and 2RUI) suggest distinct mechanisms of substrate binding (Fig. S8). In saSrtA, the β7–β8 loop is ordered in both states but moves upon ligand binding (Fig. S8*A*). In contrast, a unique N-terminal appendage in baSrtA regulates active site accessibility, as previously described, and the β7–β8 loop transitions from a disordered-to-ordered state upon substrate binding (Fig. S8*B*) (33). In both proteins, however, binding of ligand corresponds to a shift in the position of the β7–β8 loop such that it is located much closer to the β4–β5 loop (Fig. 7).

In analyses of the previously published baSrtA structures, the authors of this work mention that the β4–β5^+2^/β4–β5^+3^ positions (for baSrtA, these are β4–β5^+2^ M128 and β4–β5^+3^ S129) play a role in stabilizing a hydrophobic residue, the β7–β8^+3^ V190, upon ligand binding (Fig. 7) (33). The corresponding residues in saSrtA are F64, I65, and Y129, respectively, which would also enable a favorable hydrophobic interaction (Fig. 7). The lmSrtA structure (PDB ID: 5HU4) also shows a similar interaction: β4–β5^+2^ M129 and β4–β5^+3^ R130 with P191 in the β7–β8^+3^ position (note: M129 is the full-length protein numbering according to UniProt ID SRTA_LISMO, but is M58 in 5HU4, R130 is R59, and P191 is P120), in the unbound state (Fig. 7). Finally, our spSrtA model suggests a potential interaction between the β4–β5^+2^ I63 and β4–β5^+3^ F64 residues with β7–β8^+3^ L210 (Fig. 7).

In agreement with this proposed interaction between the β7–β8 and β4–β5 loops, we observed that our L210A spSrtA mutant reduced the activity of the protein by 46%, 59%, and 77% for the A-, G-, and S-containing peptides, respectively (Fig. 6*A*). While the alanine methyl side chain retains the hydrophobic character of the WT leucine, we speculate that its reduced size is insufficient to space the gap between the β7–β8 and β4–β5 loops, and thus this critical interaction is disrupted. Along these lines, we expressed, purified, and tested an L210P spSrtA mutant, which substitutes L210 with the proline residue found in spSrtA_monocytogenes_, a chimeric protein which was essentially inactive in our hands (Fig. S4*A*). In our substrate assay, this mutant produced results identical to those of L210A, further supporting the importance of a specific interaction between the β7–β8^+3^ residue and β4–β5 loop for sortase activity (Fig. 6*A*).

## Discussion

Although target sequence recognition by *S. aureus* SrtA is rigidly selective for a P1′ glycine, this is not true of all class A sortases, such as those from *S. pneumoniae* and *S. pyogenes* (10, 14, 29, 34). Building from our previous work, in which spSrtA was found to accept peptides containing Gly, Ala, and Ser and other residues at P1′, we have shown here that this broadened substrate scope can be attributed to the sequence of the β7–β8 loop (26). Moreover, variations in β7–β8 loop sequences can substantially impact the overall enzyme activity, affording chimeric sortases that outperform their WT counterpart *in vitro*. Together with others, the present study implicates all of the variable loops in class A sortases as being important determinants of enzyme function (12, 18, 21, 33).

With respect to structure, we propose three interactions that are facilitated by residues in the β7–β8 loop of spSrtA. They are as follows: (1) an intraloop hydrogen bond that positively affects catalytic efficiency, typically mediated by a threonine residue at the β7–β8^+6^ or β7–β8^+7^ position, (2) an interaction that hinders enzyme activity between the β7–β8 loop and β6^−2^ residues, and (3) a positive interaction between the β7–β8^+3^ and β4–β5^+2^/β4–β5^+3^ residues, typically of hydrophobic nature. Notably, there appear to be other features in this structurally conserved loop that are unique to certain class A sortases. These include the W194 residue of saSrtA, which specifically interacts with the P1 position of the CWSS and likely acts as a selectivity filter, based on our W194A spSrtA_aureus_ data (Fig. 3*A*) (19). Others identified a disordered-to-ordered transition of the baSrtA β7–β8 loop, as well as regulation by an N-terminal appendage, although more research is needed to determine whether or not this is shared by other class A sortases (33). Furthermore, while the spSrtA β7–β8 loop is seven residues in length, several class A sortase loops, for example, those of saSrtA, baSrtA, and lmSrtA studied here, are longer than seven residues. Stabilizing interactions mediated by backbone atoms likely vary in loops of differing lengths, a characteristic that was not studied in detail here. Future work is also needed to assess how the loop length influences the identified interactions described here in the context of sortase A enzymes, as well as other position-specific interactions found in other sortase classes.

In addition to informing our fundamental understanding of sortase substrate recognition, this work also has implications for the further development of SML as a protein engineering tool (3, 35). Through exchange of β7–β8 loop residues between class A sortases, we have generated chimeras such as spSrtA_faecalis_ and spSrtA_lactis_, with measurable activity against peptides possessing 15 of the 20 amino acids at P1′. Notably, stability or minor changes in the folded structures between variants were not determined and these could manifest as some of the differences in enzyme function observed. With additional development, each of these sortase chimera/substrate combinations potentially offers a new handle for *in vitro* SML applications. While preliminary attempts here to modify a protein target displaying an LPAT**V** sequence using spSrtA_faecalis_ were unsuccessful, we consider it likely that optimization of the placement of the LPAT**V** site may restore reactivity. This includes examination of the accessibility requirements for the LPAT**V** sequence and assessment of the impact of residues N- or C-terminal to the core LPAT**V** motif. Similar factors are known to affect the success of SML reactions with the widely used saSrtA/LPXT**G** system (36, 37, 38) and may need to be evaluated for our chimeras.

If successful, the development of these new sortase/substrate pairs has exciting consequences for SML engineering efforts: (1) it increases options for dual-labeling single proteins or multiplexed labeling of multiple proteins in the same systems (11, 39, 40), and (2) it may reduce the need to mutate naturally occurring protein sequences to render their termini compatible with SML. For example, using our previously published program, *MotifAnalyzer*, we found 190 instances of LPXT**G** in 189 unique proteins in the human proteome. However, if the P1′ position is now flexible, this number becomes 3606 instances of LPXT**X** in 2930 unique proteins (41).

**Figure 8:**
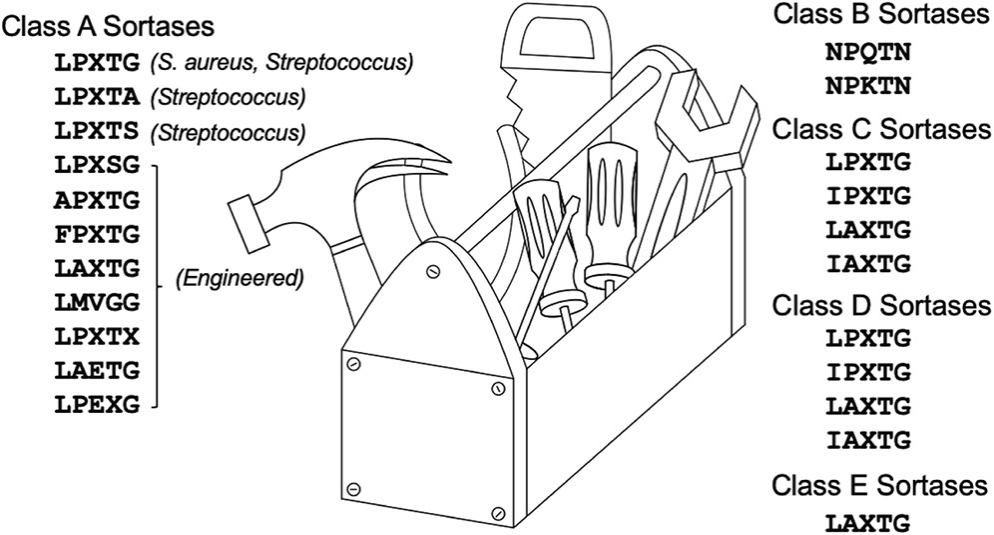
**Building a sortase toolbox for SMLexperiments**. Work from ourselves and others can be used to create a sortase “toolbox” for SML experiments, taking advantage of the various sequence motifs, both endogenous and engineered. Recognition sequences for various sortase subclasses are described in (5, 11, 12, 13, 14, 17, 22). SML, sortase-mediated ligation.

Finally, the three variable loops mentioned here (β4–β5, β6–β7, and β7–β8) are conserved in all classes of sortases (Fig. 1*B*), and previous work determining and engineering sortase selectivity of different classes, for example, sortase B, suggests similar roles for these loops in substrate recognition (5, 18, 42). Developing a deeper understanding of how residues in these loops affect substrate selectivity in all sortase classes may enable dramatic expansion of the sortase “toolbox” (Fig. 8), potentially allowing the development of ligases that are tailored to the needs of specific protein targets while also limiting off-target effects (5, 11, 12, 13, 14, 17, 22). In the over 20 years since saSrtA was discovered, the sortase superfamily has proven to be both a workhorse for protein engineering efforts and an exciting system for future discoveries and insight into the stereochemistry and mechanisms of target recognition.

## Experimental procedures

### Protein expression and purification

WT spSrtA and saSrtA proteins were expressed and purified as previously described (22). All other constructs, including chimeric and mutant proteins, were purchased from GenScript in the pET28a(+) vector. In general, protein expression and purification protocols were very similar to those previously described (22). Briefly, plasmids were transformed into *E. coli* BL21 (DE3) competent cells and grown in LB media, with protein induction at *A*_600_ 0.6 to 0.8 using 0.15 M IPTG for 18 to 20 h at 18 °C.

After cell harvest in the lysis buffer [0.05 M Tris, pH 7.5, 0.15 M NaCl, and 0.0005 M EDTA], the protein was purified using a 5-ml HisTrap HP column (GE Life Sciences, now Cytiva), using wash [0.05 M Tris, pH 7.5, 0.15 M NaCl, 0.02 M imidazole, pH 7.5, and 0.001 M TCEP] and elution [wash buffer, with 0.3 M imidazole, pH 7.5] buffers. SEC was conducted using a HiLoad 16/600 Superdex 75 column (GE Life Sciences, now Cytiva) in the SEC running buffer (0.05 M Tris, pH 7.5, 0.15 M NaCl, and 0.001 M TCEP). Purified protein corresponding to the monomeric peak was concentrated using an Amicon Ultra-15 Centrifugal Filter Unit (10,000 NWML) and analyzed by SDS-PAGE and analytical SEC (Figs. S1 and S6). Protein not immediately used was flash-frozen in the SEC running buffer and stored at −80 °C.

### Peptide synthesis

Detailed synthetic procedures are provided in the supporting information. Briefly, all peptides were synthesized *via* manual Fmoc solid-phase peptide synthesis. Peptides were synthesized either individually or in tandem using Fmoc Rink amide MBHA resin or SynPhase lantern solid supports. All other materials, including suitably protected Fmoc amino acids, and reagents for coupling, deprotection, and resin cleavage were obtained from commercial sources and used without further purification. All peptides were purified using RP-HPLC, and their identities were confirmed *via* ESI-MS. Before use in sortase-catalyzed transacylation reactions, each purified peptide was prepared as a concentrated stock solution in DMSO and/or water (see supporting information for details).

### Fluorescence assay for sortase activity

Reactions were performed in a Costar round-bottom, black, 96-well plate at a 100-μl reaction volume under the following conditions: 5 μM sortase, 50 μM peptide substrate, and 5 mM hydroxylamine nucleophile. All reactions contained 10% (*v/v*) 10× sortase reaction buffer (500 mM Tris, pH 7.5, 1500 mM NaCl, and 100 mM CaCl_2_). Reactions also contained residual DMSO from the peptide stock solutions (0.5–1.5% (*v/v*), with the exception of the Phe- and Val-containing peptides at 5%). The peptides containing phenylalanine or valine required 5% (*v/v*) DMSO for solubility under the reaction conditions. 1 mM TCEP was also included in reactions utilizing the Abz-LPATCG-K(Dnp) substrate. Reactions were initiated by the addition of the sortase enzyme, which were prepared as 10× stock solutions in 50 mM Tris, pH 7.5, 150 mM NaCl, and 1 mM TCEP. Microplates were analyzed using a BioTek Synergy H1 plate reader. The fluorescence intensity of each well was measured at 2-min time intervals over a 2-h period at RT (λ_ex_ = 320 nm, λ_em_ = 420 nm, and detector gain = 75). All reactions were performed at least in triplicate, and all of the raw fluorescence data utilized in this study are provided in Table S2. For each substrate sequence, the background fluorescence of the intact peptide in the absence of enzyme was subtracted from the observed experimental data. Background-corrected fluorescence data were then normalized to the fluorescence intensity of a benchmark reaction between WT saSrtA and Abz-LPATGG-K(Dnp) (Fig. S2*A*).

### HPLC and LC-MS characterization of sortase-catalyzed reactions

Select pairings of sortase enzyme (5 μM or 10 μM for the X-NH_2_ reactions), substrate (50 μM), and nucleophile (5 mM H_2_NOH or X-NH_2_) were repeated in the presence or absence of Ca^2+^ under reaction conditions that were otherwise identical to those described above for the fluorescence assay. These reactions were then analyzed using a Dionex UltiMate 3000 HPLC system interfaced with an Advion CMS expression mass spectrometer. Separations were achieved with a Phenomenex Kinetex 2.6 μM C18 100 Å column (100 × 2.1 mm) (aqueous [95% water, 5% MeCN, 0.1% formic acid]/MeCN [0.1% formic acid] mobile phase at 0.3 ml/min, method: hold 10% MeCN for 0.0–0.5 min, linear gradient of 10–90% MeCN for 0.5–7.0 min, hold 90% MeCN for 7.0–8.0 min, linear gradient of 90–10% MeCN for 8.0–8.1 min, re-equilibrate at 10% MeCN for 8.1–13.25 min]).

### Sequence and structural analyses

All sequences were downloaded from either the NCBI database or UniProt, as indicated (43, 44, 45). Sequence alignments were performed using MAFFT, T-Coffee, or BlastP (23, 46, 47). Visualization of our T-Coffee alignment was done using BoxShade. ConSurf analyses were performed using the online server, with 2KID as a template and insertion or our own multiple sequence alignment performed using MAFFT (24, 25). Alignments were visualized using Jalview (48). Homology modeling was performed using the SWISS-MODEL web interface (30, 31). Structural analyses and figure rendering were done using PyMOL. Enzyme assay graphs were prepared using GraphPad Prism 9.1.2.

## Data availability

All data are contained in the article and the supporting information.

## Supporting information

This article contains supporting information (22, 49, 50).

## Conflict of interests

The authors declare that they have no conflicts of interest with the contents of this article.
