# Supplementary material for: Sequence variation in the β7–β8 loop of bacterial class A sortase enzymes alters substrate selectivity
Source: J Biol Chem. 2021 Jul 22;297(2):100981. doi: 10.1016/j.jbc.2021.100981 (PMC8361268; doi:10.1016/j.jbc.2021.100981)
Supplement: Supporting information [file mmc1.pdf]

Sequence variation in the β7-β8 loop of bacterial Class A sortase enzymes alters substrate selectivity

Isabel M. Piper, Sarah A. Struyvenberg, Jordan D. Valgardson, D. Alex Johnson, Melody Gao, Katherine Johnston, Justin E. Svendsen, Hanna M. Kodama, Kelli L. Hvorecny, John M. Antos, Jeanine F. Amacher

Table of Contents

Fig S1. Representative analytical SEC chromatograms of sortase preparations following IMAC and preparative SEC 2

Table S1. LC-MS characterization of synthetic peptides and reaction products 3

Fig S2. Sample benchmark reaction data and HPLC analysis of model reactions 4

Fig S3. SaSrtA active site including W194 residue 5

Fig S4. Additional biochemical data for chimeric spSrtA proteins 6

Fig S5. Comparison of $spSrtA_{faecalis}$ substrate selectivity trends (HPLC vs fluorescence) 7

Fig S6. Representative analytical SEC chromatograms of spSrtA loop mutants following IMAC and preparative SEC 8

Fig S7. The homology model of spSrtA is very similar to other *Streptococcus* Class A structures 9

Fig S8. Structural characteristics of the β7-β8 loops of Class A sortases. 10

Supplemental experimental procedures for peptide synthesis 11-14

References 15

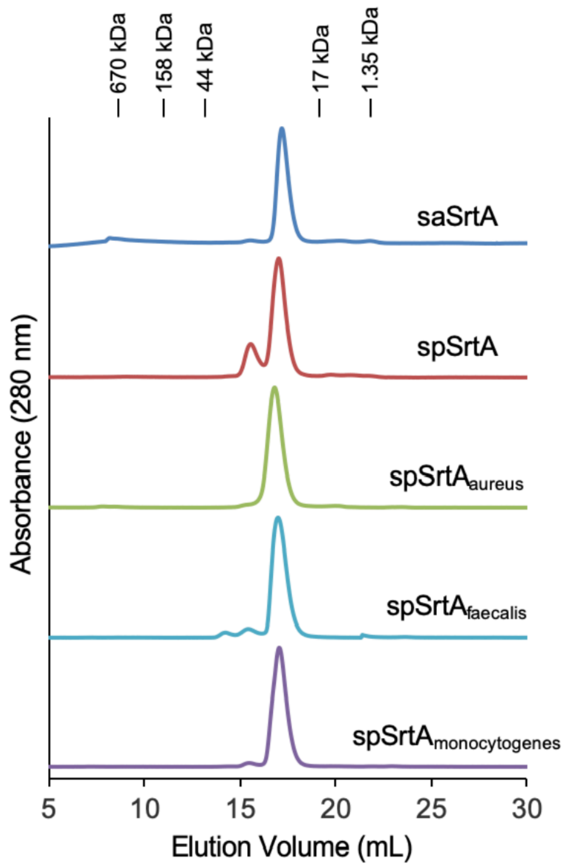

Fig. S1. Representative analytical SEC chromatograms of sortase preparations following IMAC and preparative SEC. Separations achieved using a Superdex 200 Increase 10/300 GL column (GE Life Sciences) with a mobile phase consisting of 0.05 M Tris pH 7.5, 0.15 M NaCl, 0.001 M TCEP. Elution volumes for molecular weight standards (Bio-Rad) are indicated above the chromatograms.

Table S1. Mass spectrometry (LC-MS) characterization of synthetic peptides and relevant products from *in vitro* sortase-catalyzed model reactions.[a] Residue substitutions as compared to the canonical CWSS (LPXTG) are highlighted in red.

Peptide	Mass (m/z)	
	calculated	observed
Abz-LPATAG-K(Dnp)	941.4	941.5
Abz-LPATCG-K(Dnp)	973.4	973.3
Abz-LPATDG-K(Dnp)	985.4	985.5
Abz-LPATEG-K(Dnp)	999.5	999.4
Abz-LPATFG-K(Dnp)	1017.5	1017.5
Abz-LPATGG-K(Dnp)	927.4	927.5
Abz-LPATHG-K(Dnp)	1007.5	1007.4
Abz-LPATIG-K(Dnp)	983.5	983.5
Abz-LPATKG-K(Dnp)	998.5	998.6
Abz-LPATLG-K(Dnp)	983.5	983.6
Abz-LPATMG-K(Dnp)	1001.4	1001.4
Abz-LPATNG-K(Dnp)	984.5	984.5
Abz-LPATPG-K(Dnp)	967.5	967.6
Abz-LPATQG-K(Dnp)	998.5	998.4
Abz-LPATRG-K(Dnp)	1026.5	1026.5
Abz-LPATSG-K(Dnp)	957.4	957.3
Abz-LPATTG-K(Dnp)	971.5	971.5
Abz-LPATVG-K(Dnp)	969.5	969.4
Abz-LPATWG-K(Dnp)	1056.5	1056.6
Abz-LPATYG-K(Dnp)	1033.5	1033.4
Abz-LPAT-NHOH	535.3	535.3[b]
Abz-LPATG-NH_2	590.3	590.3[b]
Abz-LPATA-NH_2	606.3	606.3[b]
Abz-LPATS-NH_2	618.4	618.4[b]
AG-K(Dnp)	440.2	440.2[b]
FG-K(Dnp)	516.2	516.2
GG-K(Dnp)	426.2	426.2[b]
IG-K(Dnp)	482.2	482.2
LG-K(Dnp)	482.2	482.2
MG-K(Dnp)	500.2	500.1
NG-K(Dnp)	483.2	483.2
SG-K(Dnp)	456.2	456.2[b]
VG-K(Dnp)	468.2	468.2
WG-K(Dnp)	555.2	555.2
YG-K(Dnp)	532.2	532.2
G-K(Dnp)	369.2	369.2[b]

[a]Calculated and observed masses represent $[M+H]^+$ ions (monoisotopic). [Abz = 2-aminobenzoyl fluorophore, Dnp = 2,4-dinitrophenyl chromophore, -NHOH = hydroxamic acid at C-terminus, $-NH_2$ primary amide at C-terminus]. [b]Product observed in multiple independent *in vitro* sortase-catalyzed model reactions. In all cases the observed m/z was within ±0.1 of the calculated m/z.

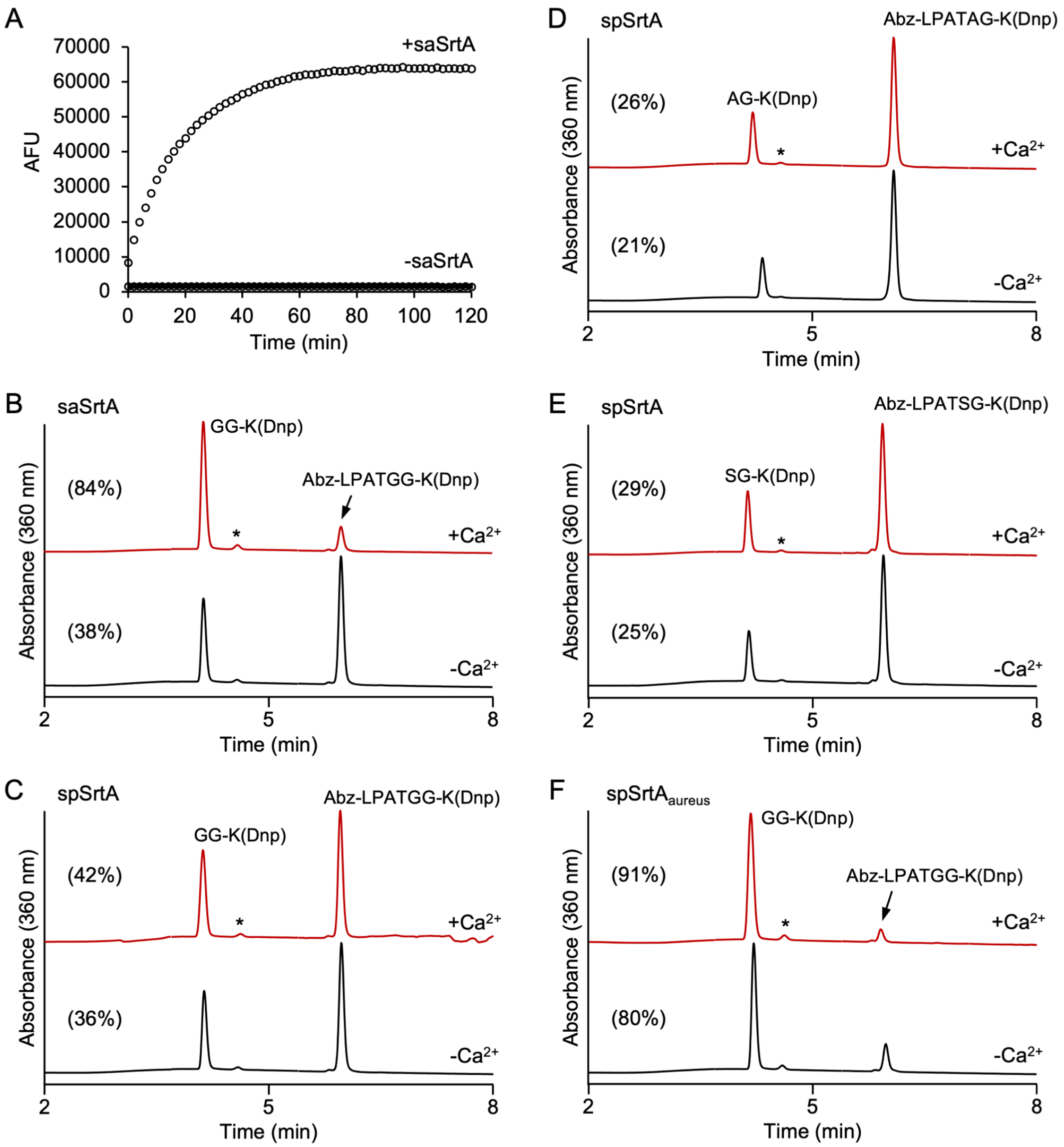

Fig S2. Sample benchmark fluorescence data and HPLC characterization of select transacylation reactions in the presence and absence of Ca^{2+}. (A) Representative fluorescence data (λ_{ex} = 320 nm, λ_{em} = 420 nm) for the reaction of Abz-LPATGG-K(Dnp) and H_2NOH in the presence and absence of saSrtA. The benchmark AFU value used for scaling the majority of fluorescence data for other enzyme/substrate pairings was determined from five independent experiments. A benchmark AFU value derived from three additional, independent saSrtA/Abz-LPATGG-K(Dnp) reactions was used for scaling the fluorescence data in **Figs. 3A, 4, 6A, S4B**. (B-F) HPLC analyses of sortase-catalyzed reactions between Abz-LPATXG-K(Dnp) and H_2NOH confirmed that the activity of saSrtA was Ca^{2+}-dependent (panel A), whereas spSrtA and $spSrtA_{aureus}$ did not require Ca^{2+} (panels B-F). For all chromatograms, estimated substrate conversion at the 2 h timepoint is shown in parentheses. All peaks identities were confirmed via LC-MS (**Table S1**), and * denotes the position of the Abz-LPAT-NHOH ligation product. Low peak intensity is expected for this species due to the minimal absorbance of the Abz fluorophore at 360 nm.

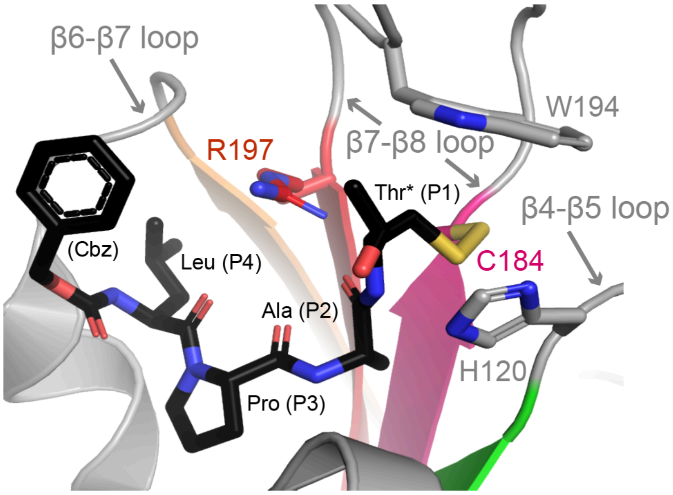

Fig S3. SaSrtA active site including W194 residue. The active-site structure of *S. aureus* SrtA (saSrtA) is shown in cartoon representation as in **Fig. 1A**, with β-strands colored and labeled (PDB ID 2KID) (16). The side chains of the catalytic residues (H120, C184, and R197) are shown as sticks, colored by heteroatom (O=red, N=blue, S=yellow), and labeled. The peptide analog, Cbz-LPAT*, where Cbz is a carbobenzyloxy protecting group and T* is (2*R*,3*S*)-3-amino-4-mercapto-2-butanol, is shown as black sticks and colored by heteroatom (16). The variable loops are labeled and indicated by gray arrows. Here, W194 is also shown with side chain sticks in gray and colored by heteroatom.

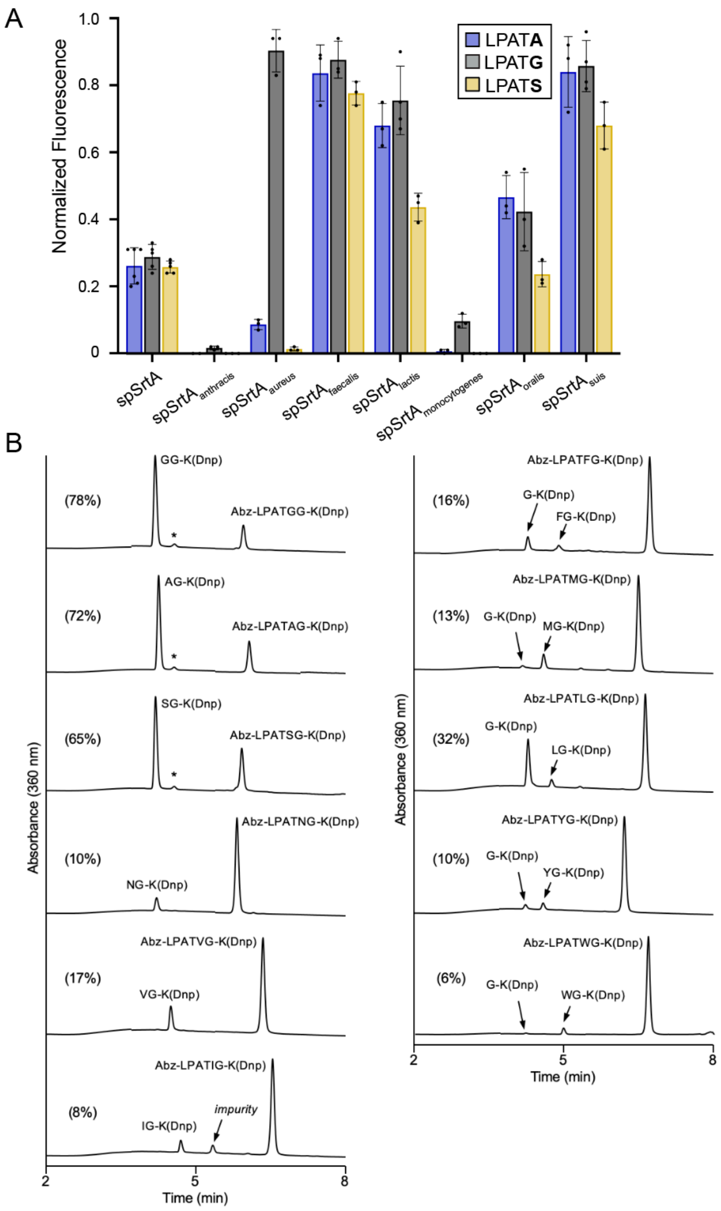

Fig S4. Additional biochemical data for chimeric spSrtA proteins. (A) spSrtA chimeras with different β7-β8 loop sequences exhibit varied activity against a small panel of LPAT**X** substrates. (B) HPLC analyses of select reactions between Abz-LPATXG-K(Dnp), H_2NOH, and $spSrtA_{faecalis}$ in the absence of Ca^{2+} reveal single cleavage sites for certain substrates (X = G, A, S, N, V, I) and a mixture of cleavage products for others (X = F, M, L, Y, W). Similar variations in cleavage sites have been observed previously for wild-type spSrtA (1). For all chromatograms, overall substrate conversion at the 2 h timepoint is shown in parentheses. All peaks identities were confirmed via LC-MS (**Table S1**), and where visible * denotes the position of the Abz-LPAT-NHOH ligation product.

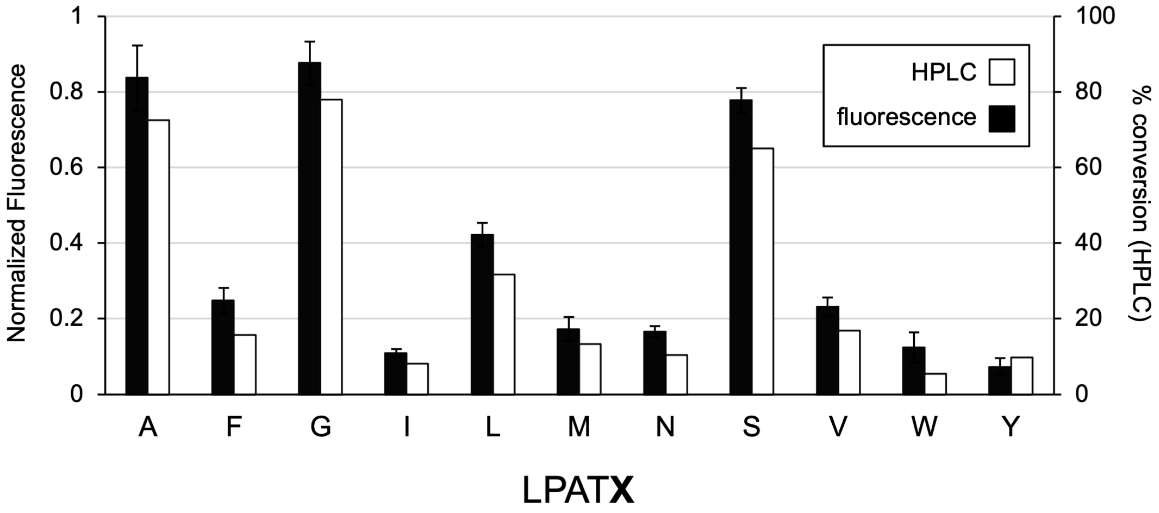

Fig S5. Comparison of substrate selectivity trends for spSrtA$_{faecalis}$ as determined via HPLC and fluorescence assay. Normalized fluorescence data (black) for the reaction of select Abz-LPATXG-K(Dnp) substrates with spSrtA$_{faecalis}$ is reproduced from **Fig. 4** in the main text, and was measured as described in Experimental Procedures. Percent substrate conversion, as determined by HPLC (white), was estimated from relevant peak areas observed in the 360 nm chromatogram. HPLC data represents single data points, while fluorescence experiments were conducted in triplicate. All data corresponds to the 2 h reaction timepoint.

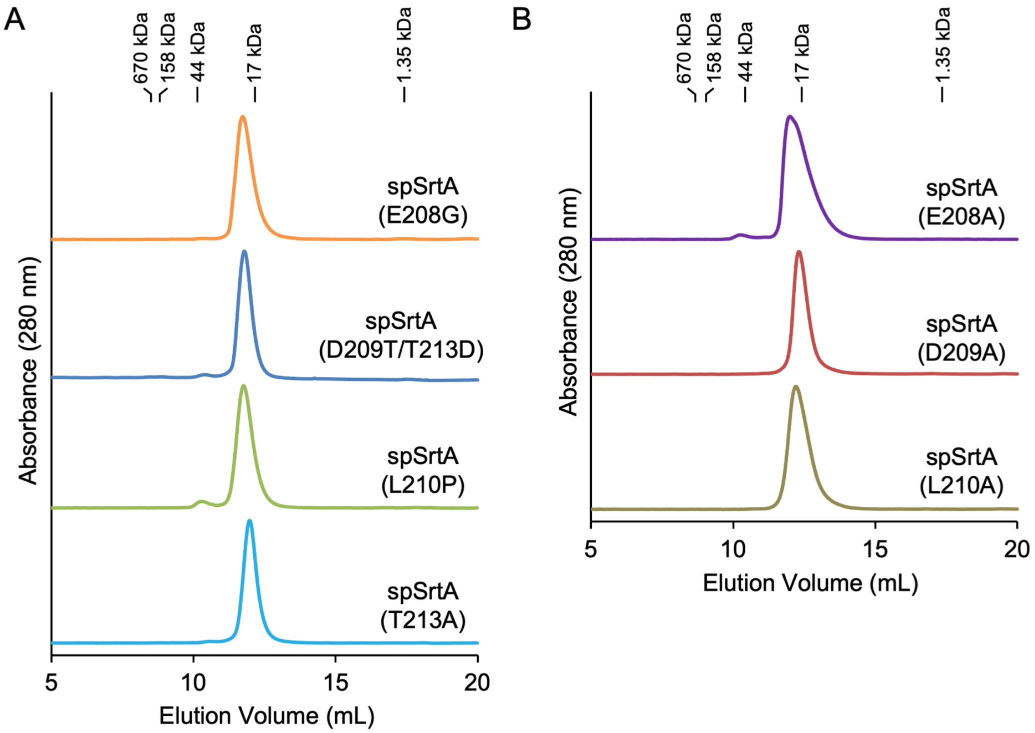

Fig. S6. Representative analytical SEC chromatograms of spSrtA loop mutants following IMAC and preparative SEC. Separations achieved using an Enrich SEC 70 column (Bio-Rad) column with a mobile phase consisting of 0.05 M Tris pH 7.5, 0.15 M NaCl, 0.001 M TCEP at (A) 0.5 mL/min or (B) 1.0 mL/min Elution volumes for molecular weight standards (Bio-Rad) are indicated above the chromatograms.

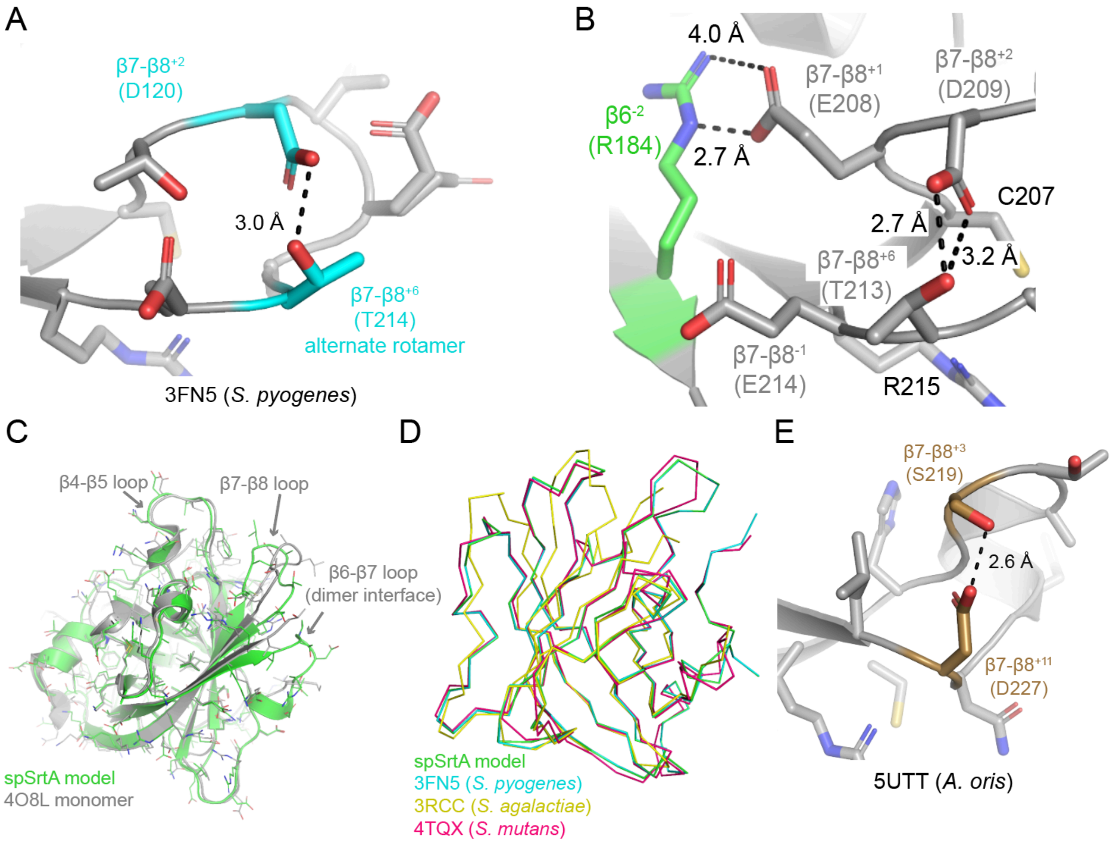

Fig. S7. The homology model of spSrtA is very similar to other *Streptococcus* Class A structures. (A) *S. pyogenes* SrtA (PDB ID 3FN5) is in gray ribbon. The β7-β8 loop side chains are in stick representation and colored by heteroatom. The residues forming a hydrogen-bond have cyan carbons and are labeled. The hydrogen bond is shown with a black dashed line, with measurements indicated. This figure shows the hydrogen-bond formed in the alternate confirmation of T214 as compared to **Fig. 6B**. (B) The spSrtA homology model was created using SwissModel and *Streptococcus pyogenes* SrtA (PDB ID 3FN5) as a template. This model (green cartoon), as well as (C) a monomer extracted from the domain-swapped dimer structure of spSrtA (4O8L, gray cartoon) are shown with the side chains as sticks and colored by heteroatom (O=red, N=blue, S=yellow). These two structures have an overall RMSD of 0.083 Å over 567 main chain atoms. Residues are perfectly aligned, with the exception of backbone variability in the β7-β8 loop and missing residues in the "4O8L monomer" β4-β5 loop, which is the location of the domain swapped region. Notably, the β7-β8 loop is also involved in the dimer interface in the 4O8L structure. (D) The ribbon traces of the spSrtA model (green), *S. pyogenes* SrtA (3FN5, cyan), *Streptococcus agalactiae* SrtA (3RCC, yellow), and *Streptococcus mutans* SrtA (4TQX, pink) are shown. Alignment of the model main chain atoms revealed overall RMSD values of 0.083 Å (567 atoms) for *S. pyogenes* SrtA, 0.773 Å (384 atoms) for *S. agalactiae* SrtA, and 0.456 Å (530 atoms) for *S. mutans* SrtA. Recall that the *S. pyogenes* SrtA structure was used as the model template, which explains the very low RMSD value. (E) The structure of *A. oris* SrtA (PDB ID 5UTT) shows a hydrogen bond formed between β7-β8^{+3} S219 and β7-β8^{+11} D227 (colored gold, with side chain sticks colored by heteroatom). The distance is 2.6 Å, as labeled. The rest of the protein is in gray cartoon with side chains in stick representation and colored by heteroatom (N=blue, O=red).

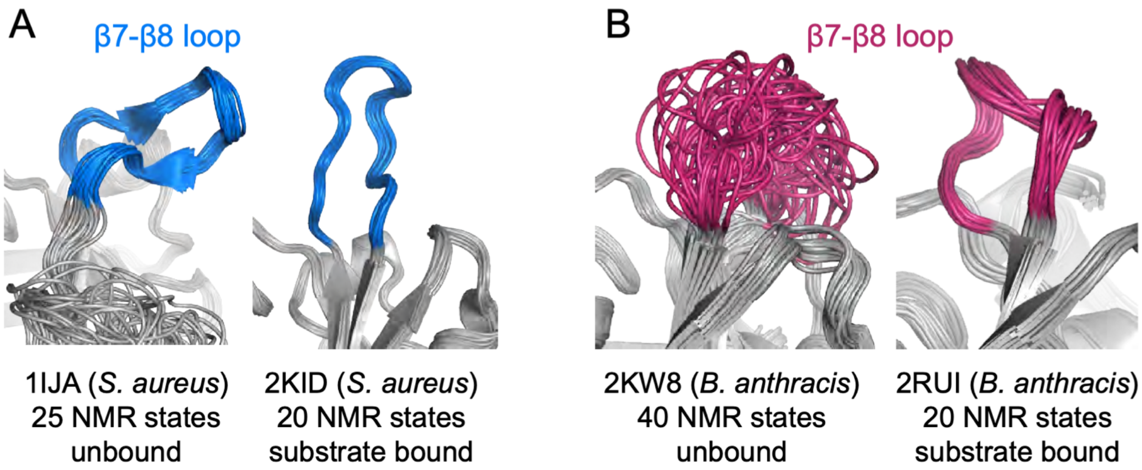

Fig. S8. Structural characteristics of the β7-β8 loops of Class A sortases. (A-B) Flexibility in the β7-β8 loop differs in Class A sortases; for *S. aureus* SrtA (A), there is little to no flexibility in the loop in the unbound structure. In contrast, NMR structures of *B. anthracis* SrtA (B) reveal a large degree of flexibility in the unbound structure (left image, PDB ID 2KW8), which is reduced upon substrate binding (right image, 2RUI). For all, the proteins are in cartoon representation and colored gray, with the exception of the residues in the β7-β8 loop: *S. aureus* SrtA (blue) and *B. anthracis* SrtA (dark pink).

Supplemental Experimental Procedures for Peptide Synthesis

General Synthetic Procedures. All peptide substrates were synthesized via manual Fmoc solid phase peptide synthesis (SPPS) using Fmoc Rink amide MBHA resin (synthesis of individual sequences) or SynPhase lanterns (tandem synthesis of multiple sequences) as the solid support. All steps (washing, coupling, deprotection) were performed at room temperature and included gentle agitation on a bench-top rocking platform. All materials, including standard Fmoc amino acids, Fmoc Rink amide MBHA resin, Mimotope SynPhase lanterns (PSLRAM015), and reagents for coupling, deprotection, and resin cleavage were obtained from commercial sources and used without further purification. Incorporation of the 2,4-dinitrophenyl (Dnp) chromophore was achieved using a commercially available lysine building block (Fmoc-L-Lys(Dnp)-OH) purchased from ApexBio. Boc-2-aminobenzoic acid was obtained from Chem-Impex International. A colorimetric ninhydrin test kit for monitoring coupling reactions was purchased from Anaspec.

Synthesis of Individual Sequences. Peptides that were independently synthesized utilized Fmoc Rink amide MBHA resin as the solid support. First, a 15 mL polypropylene synthesis vessel fitted with appropriate frits and inlet/outlet caps was loaded with Rink resin at a 0.1 mmol scale. The resin was swollen prior to synthesis with ~10 mL N-methyl-2-pyrrolidone (NMP) (3x, 10 min per wash). Following the NMP washes, the base-labile Fmoc protecting group was removed with 20% piperidine in NMP (2x, 10-20 min), followed by washing with ~10 mL of NMP (3x, 5 min per wash). For each added residue, the following coupling solution was prepared in a 3 mL glass vial: Fmoc amino acid (0.3 mmol, 3.0 equivalents relative to resin loading), O-(benzotriazol-1-yl)-N,N,N',N'-tetramethyluronium (HBTU) (0.3 mmol), N-N-diisopropylethylamine (DIPEA) (0.5 mmol) dissolved in 3 mL of NMP. Following thorough mixing, this solution was added to the

synthesis vial with the deprotected resin along with ~3 mL of additional NMP to fully suspend the resin. Couplings were incubated for a minimum of 1 hour at room temperature. Following each coupling, the resin was washed with ~10 mL NMP (3x, 10 min per rinse). The resin was then deprotected with ~10 mL of 20% piperidine in NMP (2x, 10-20 min per treatment), and washed with ~10 mL NMP (3x, 5 min per wash). Repeated cycles of coupling and deprotection were then used to assemble the target sequence. An aminobenzoyl (Abz) fluorophore was installed at the N terminus of all peptides through the coupling of Boc-2-aminobenzoic acid using the same coupling protocol described above. Following the synthesis of the desired sequence, the resin was washed with ~10 mL of NMP (3x, 10 min per wash), followed by ~10 mL of dichloromethane (DCM) (3x, 10 min per wash). A 5 mL solution of 95:2.5:2.5 TFA/TIPS/H_2O was used to cleave most peptide from the resin (2x, 30 min per treatment). Peptides containing cysteine or methionine were cleaved using a solution of 90:2.5:2.5:5 TFA/TIPS/EDT/thioanisole. Peptides containing tryptophan required a cleavage solution of 88:5:5:2 TFA/phenol/H_2O/TIPS. The cleaved peptide solutions were concentrated via a rotary evaporator, and the remaining residue was added dropwise to 35 mL of diethyl ether chilled over dry ice. The suspension was then centrifuged at 4500 rpm for 5 minutes at 4° C to collect the precipitated peptide. The diethyl ether was decanted and the crude peptide was dried overnight under vacuum.

Tandem Peptide Synthesis. SynPhase lanterns (0.015 mmol loading capacity) were used in order to discretely synthesize numerous peptides in tandem. For parallel coupling of the same residue, multiple lanterns were loaded into a single 15 mL polypropylene synthesis vessel, and deprotection and rinsing were carried out in the same manner and volume as described above for individual peptide sequences. Coupling solutions for attaching Fmoc amino acids were also prepared

similarly, with at least a 3x molar excess of the Fmoc amino acid and HBTU, and at least a 5x molar excess of DIPEA. The volume of NMP was adjusted according to the number of lanterns used in order to maintain reagent concentrations consistent with those used in the synthesis of individual peptide sequences. In order to couple the residues that varied between the peptides, the lanterns were transferred from the 15 mL synthesis vessel to individual 3 mL glass vials containing 1 mL of the appropriate coupling solution. Couplings were incubated for at least 2 hours and were not agitated. Prior to being returned to the synthesis vessel, the lanterns were washed with ~3 mL of NMP (3x, 5 min per wash) to prevent cross-contamination of the coupling solution. A fourth wash with ~10 mL NMP was carried out once the lanterns were transferred back to the original synthesis vessel. Once the peptides were complete, each lantern was moved to individual 3 mL vials for cleavage with 1 mL of 95:2.5:2.5 TFA/TIPS/H_2O (2x, 30 min per treatment). Peptides containing cysteine or methionine were cleaved using a solution of 90:2.5:2.5:5 TFA/TIPS/EDT/thioanisole. The cleaved peptide solution was then concentrated using a rotary evaporator. The remaining residue was then added dropwise to a 15 mL polypropylene centrifuge tube containing 2 mL of dry ice-chilled diethyl ether. The majority of sequences precipitated under these conditions and were recovered via centrifugation (4500 rpm for 10-20 minutes at 4 ^{o}C). The diethyl ether was then decanted and the crude peptides were dried overnight under vacuum. In cases where the peptides were not effectively precipitated from ether, they were suspended in ~10 mL of water and lyophilized.

Peptide Purification. Crude peptides from both independent and tandem synthesis were resolvated in a minimum volume of MeCN and H_2O and were purified via RP-HPLC [Phenomenex Luna 5 μm, 100 Å C18 column (10 x 250 mm), aqueous (95:5 H_2O/MeCN, 0.1% formic acid) / MeCN

(0.1% formic acid) mobile phase at 4.0 mL/min, method: hold 20% MeCN (0.0-2.0 min), linear gradient of 20-90% MeCN 2.0-15.0 min, hold 90% MeCN 15.0-17.0 min)]. The purified peptide fractions were concentrated via a rotary evaporator and then lyophilized. The identity of each peptide was confirmed via ESI-MS (**Table S1**), and the purity of each peptide was confirmed to be >90% by RP-HPLC.

Peptide Stock Solution Preparation. Prior to use in sortase-catalyzed reactions, purified peptides were dissolved in a minimum volume of 10:90 DMSO/H_2O, 1:1 DMSO/H_2O, or pure DMSO. Peptide concentrations were estimated using the absorbance of the Dnp chromophore at 360 nm (extinction coefficient = 17,300 $M^{-1}cm^{-1}$) (2, 3). The stocks were then diluted to a working concentration of either 1 mM, 5 mM, or 10 mM depending on DMSO content in order to ensure that the final sortase-catalyzed reaction mixtures contained ≤5% DMSO by volume. Specifically, reactions utilizing the Abz-LPATFG-K(Dnp) substrate contained a final DMSO concentration of 5% (v/v), whereas reactions with the remaining peptides substrates contained ~0.5-1.5% DMSO (v/v).

References

1. Nikghalb KD, Horvath NM, Prelesnik JL, Banks OGB, Filipov PA, Row RD, et al. Expanding the Scope of Sortase-Mediated Ligations by Using Sortase Homologues. ChemBioChem. 2018;19(2):185-95.
2. Johanning K, Juliano MA, Juliano L, Lazure C, Lamango NS, Steiner DF, et al. Specificity of prohormone convertase 2 on proenkephalin and proenkephalin-related substrates. J Biol Chem. 1998;273(35):22672-80.
3. Bennett NR, Jarvis CM, Alam MM, Zwick DB, Olson JM, Nguyen HV, et al. Modular Polymer Antigens To Optimize Immunity. Biomacromolecules. 2019;20(12):4370-9.
